# DNA-origami-directed virus capsid polymorphism

**DOI:** 10.1038/s41565-023-01443-x

**Published:** 2023-07-17

**Authors:** Iris Seitz, Sharon Saarinen, Esa-Pekka Kumpula, Donna McNeale, Eduardo Anaya-Plaza, Vili Lampinen, Vesa P. Hytönen, Frank Sainsbury, Jeroen J. L. M. Cornelissen, Veikko Linko, Juha T. Huiskonen, Mauri A. Kostiainen

**Affiliations:** 1 Department of Bioproducts and Biosystems, Aalto University, Aalto, Finland; 2 Institute of Biotechnology, Helsinki Institute of Life Science HiLIFE, University of Helsinki, Helsinki, Finland; 3 Centre for Cell Factories and Biopolymers, Griffith Institute for Drug Discovery, Griffith University, Nathan, Queensland Australia; 4 Faculty of Medicine and Health Technology, Tampere University, Tampere, Finland; 5 Department of Molecules and Materials, MESA+ Institute for Nanotechnology, University of Twente, Enschede, Netherlands; 6 LIBER Center of Excellence, Aalto University, Aalto, Finland; 7 Institute of Technology, University of Tartu, Tartu, Estonia

**Keywords:** Nanostructures, Nanoparticles, Nanobiotechnology

## Abstract

Viral capsids can adopt various geometries, most iconically characterized by icosahedral or helical symmetries. Importantly, precise control over the size and shape of virus capsids would have advantages in the development of new vaccines and delivery systems. However, current tools to direct the assembly process in a programmable manner are exceedingly elusive. Here we introduce a modular approach by demonstrating DNA-origami-directed polymorphism of single-protein subunit capsids. We achieve control over the capsid shape, size and topology by employing user-defined DNA origami nanostructures as binding and assembly platforms, which are efficiently encapsulated within the capsid. Furthermore, the obtained viral capsid coatings can shield the encapsulated DNA origami from degradation. Our approach is, moreover, not limited to a single type of capsomers and can also be applied to RNA–DNA origami structures to pave way for next-generation cargo protection and targeting strategies.

## Abstract (web summary)

DNA and RNA origami nanostructures direct the size, shape and topology of different virus capsids in a user-defined manner while shielding encapsulated origamis from degradation.

## Main

Protein cages can be prepared by de novo design, by engineering of existing proteins or by isolating them from nature. Design examples of non-native systems include metal-coordinated cages^1,2^, and single- and two-component icosahedral architectures^3,4^. Such systems have proven to be effective in, for example, immunogen display and potent vaccine design^5^. Moreover, native virus capsids have unique assembly properties, making them popular building blocks within nanobioengineering^6–10^. Polymorphic behaviour has been observed upon in vitro reassembly for several virus types^11–14^; however, directing the polymorphism in a user-defined way remains challenging^15,16^.

The spherical cowpea chlorotic mottle virus (CCMV, diameter *d* = 28 nm) is one of the most studied viruses. Its 180 capsid protein (CP) copies are arranged into 20 hexamers and 12 pentamers, resulting in a quasi-icosahedral *T* = 3 symmetry^17^. Its reversible assembly process is well characterized and highly dependent on environmental conditions such as pH and ionic strength. Due to the CPs’ innate protein assembly pathways, reassembly results in the formation of hexagonal sheets, empty spheres and tubes^18–21^. Although control over spherical assemblies has been demonstrated by encapsulation of both organic and inorganic materials^22–24^, the formation of other assemblies that deviate from the native icosahedral or tubular symmetry cannot be achieved in a modular way.

**Fig. 1:**
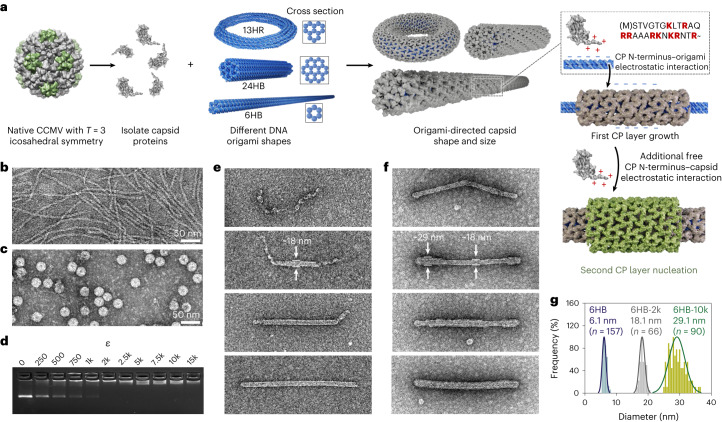
Formation of capsid-coated DNA origami structures. **a**, CPs are isolated from native CCMV (left) and complexed with different DNA origami shapes, resulting in a coating (middle) whose properties are determined by the origami structure. The assembly is driven by electrostatic (positively charged amino acids in the N-terminus marked in red) and protein–protein interactions (right). A second protein layer can develop on top of the first one due to electrostatic interactions between the N-terminus and the negatively charged parts of the CP surface. **b**, Negative-stain TEM image of plain 6HB structures. **c**, Negative-stain TEM image of native CCMV particles. **d**, EMSA shows electrophoretic mobility decrease of 6HB upon complexation with CPs when increasing *ε*. **e**, Development of a single layer of CPs on the 6HB origami template using *ε* ≤ 2k. **f**, Subsequent development of a second CP layer on top of **e**. **g**, Observed size distributions (in diameter) for plain 6HB (blue) and 6HB complexed at *ε* = 2k (grey) or at *ε* = 10k (green). The image width of all TEM images corresponds to 500 nm. Source data

In this article we utilize DNA origami^25^ templates to obtain precise control of the virus capsid assembly’s size and shape. To this end, a long, single-stranded DNA scaffold strand is assembled into well-defined two- and three-dimensional structures^26,27^ through programmable hybridization with short, single-stranded staple sequences^25,28^. First we study directing the assembly of CCMV CPs using a six-helix bundle (6HB) DNA origami (Fig. 1). 6HB fits inside the previously described, hollow tubes^18^ (considering also the thickness of the CP layer^17^), and additionally, it closely mimicks the packaging of DNA observed in naturally occurring viral systems^29^. By optimizing the ratio between DNA origami and CPs, complexes with multiple protein layers were obtained and further characterized using single-particle cryo-electron microscopy (cryo-EM) reconstruction. This approach allows not only the encapsulation of non-linear, non-tubular and RNA–DNA hybrid structures, but is also applicable to other virus species, such as polyoma viruses. Furthermore, it presents a versatile technique for protecting DNA against nuclease degradation, thus making it attractive for vaccine and nucleic acid delivery vector development.

## Forming CP–DNA origami complexes

We have used five different DNA origami structures to investigate the properties and possible geometric limitations that govern the assembly of CCMV CPs. 6HB and 24HB are cylindrical structures with diameters (*d*) of 6 nm and 14 nm, respectively, whereas the 13-helix ring (13HR) is a toroidal structure, the 60HB is a brick-like object and the nanocapsule is a stimuli-responsive object (Supplementary Note 1). The complexation process is driven by protein–protein interactions and by electrostatic interactions between the negatively charged phosphate backbone of the DNA origami and the positively charged amino acid (aa) residues (+9) in the 26-residue arginine-rich N-terminal region of the protein (190 aa, ~20 kDa) (Fig. 1a). The complexation process was found not to be limited to a single protein layer (grey), but rather the positively charged CP residues interacted also with negatively charged patches on the CPs’ surface, resulting in the nucleation of a second protein layer (green).

After characterizing the starting materials (dimensions are given as average (avg) ± s.d. throughout), 6HB (*d*_avg_ = 6.1 ± 0.6 nm) and intact CCMV (*d*_avg_ = 26.8 ± 1.0 nm) (Fig. 1b,c) with negative-stain transmission electron microscopy (TEM), the complexation of isolated CPs and 6HB was performed at physiological conditions (pH 7.3, 150 mM NaCl) using the CPs in excess. The excess, *ε*, is defined as the molar ratio between the protein monomer and the DNA origami (*c*_CP_/*c*_origami_). The complexation was monitored using an electrophoretic mobility shift assay (EMSA) based on agarose gel electrophoresis (AGE). With increasing *ε*, a gradual decrease in the intensity of the 6HB leading band is observed, while another band with lower electrophoretic mobility appears (Fig. 1d), corresponding to the well-known fast assembly behaviour of CCMV CPs with low cooperativity^30^. The decrease in electrophoretic mobility, stagnating from approximately *ε* = 2k, with k standing for the multiplier 1,000, that is, *ε* = 2,000, indicates a notably less negative surface charge of the origami–CP complex. Negative-stain TEM images and small-angle X-ray scattering (SAXS; Supplementary Note 2 and Supplementary Fig. 2a) for samples complexed at *ε* = 2k (6HB-2k) reveal 6HB complexes with increased diameter due to a highly ordered protein shell (Fig. 1e, bottom), which develops from nucleation sites along the origami structure (*ε* < 2k). When using *ε* > 2k, assembly of free CPs on top of the first protein layer is observed, being particularly pronounced at *ε* = 10k (Fig. 1f). Statistical analysis of the diameter of the complexed structures (Fig. 1g) shows a clear change from *d*_avg_ = 6.1 ± 0.6 nm (plain 6HB, blue) to 18.1 ± 0.9 nm (6HB-2k, single-layer coating, grey) and further to 29.1 ± 2.5 nm (6HB-10k, double-layer coating, green). 6HB is rather flexible^31^, which might contribute to both partially collapsed origami structures upon initial electrostatic binding of the CP and to the proneness to structural defects, including loops, bends and incomplete layers, which might also explain the wider size distribution for 6HB-10k (Supplementary Note 3).

In general, CCMV assembly has been described as being initiated by CP dimers forming pentamers. Adding dimeric subunits results in pentameric and hexameric capsomeres with curvature^32,33^. Nucleation^32^ followed by elongation^34^ has been suggested for larger icosahedral assemblies. However, in the absence of RNA, assembly based on dimers forming hexameric morphological units as nucleation sites has been proposed^17^. For low ionic strengths, implying stronger protein–DNA compared with protein–protein interactions, en masse mechanisms have been suggested for the assembly. We expect our protein shells to nucleate into a hexameric lattice. Previous work on the assembly of brome mosaic virus (BMV, closely related to CCMV) on spherocylindrical substrates reported a dependency between the diameter of the rod and the preferred location of nucleation along the rod^13,35^. For 6HB-500, although the length/progression of the coating is heterogeneous, nucleation is mainly observed around the middle part of the rod. In contrast, when decreasing the aspect ratio, nucleation is also observed on the spherical cap regions (Supplementary Note 4). Due to the lack of nucleation of a second protein layer at this early stage, we hypothesize that a (almost) fully developed first protein layer is required to facilitate the formation of the second one. Although the CPs’ surface has negatively charged patches, it seems to be more favourable for the CPs to interact with the highly negatively charged surface of the origami.

The geometric properties of the observed complexed structures with respect to the outer diameter of the tubes are consistent with previously reported tubes^18,21,36,37^. Whereas controllability over the assemblies’ length was previously low^36,38^, here, in contrast, a narrow length distribution of the complexed structures is obtained, suggesting the encapsulation of exactly one 6HB origami per complexed structure.

## Cryo-electron microscopy reconstruction

**Fig. 2:**
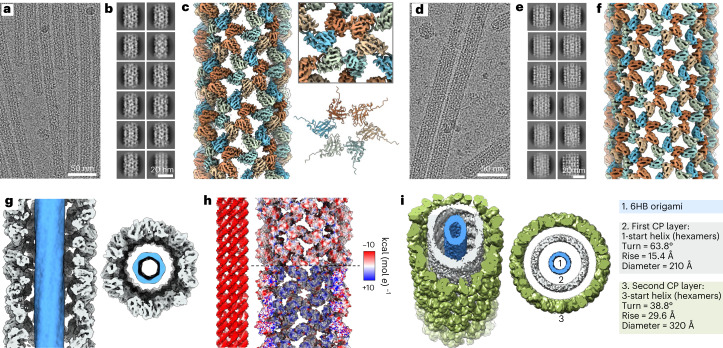
Single-particle reconstruction of complexed 6HB structures using cryo-EM. **a**, Representative micrograph image of 6HB coated with a single protein layer (6HB-2k). **b**, Selected 2D class averages for 6HB-2k. **c**, Cryo-EM density of the tube (left) and a selected hexamer (top right). In an atomic model (bottom right) the CP (PDB:1cwp) was flexibly fitted to the EM density. **d**, Representative image of the double-layered filaments formed by 6HB and the CPs (6HB-10k). **e**, Selected 2D class averages for 6HB-10k. **f**, Cryo-EM density of the outer layer of a double-layered tube. **g**, Cross section and top view of 6HB-2k complexes showing the DNA origami in blue. **h**, Electrostatic potential surface suggests a negative potential for both the DNA origami (left) and the outward-facing protein surface of the first layer (top right), whereas the DNA adjacent surface of the protein shell (bottom right) possesses a positive potential. **i**, A 3D model of the assembled double-layer structure shows the different symmetry of the two layers assembling on 6HB origami (1). While the inner layer (2) has a 1-start helix symmetry, the outer layer (3) is defined by a 3-start helix.

**Fig. 3:**
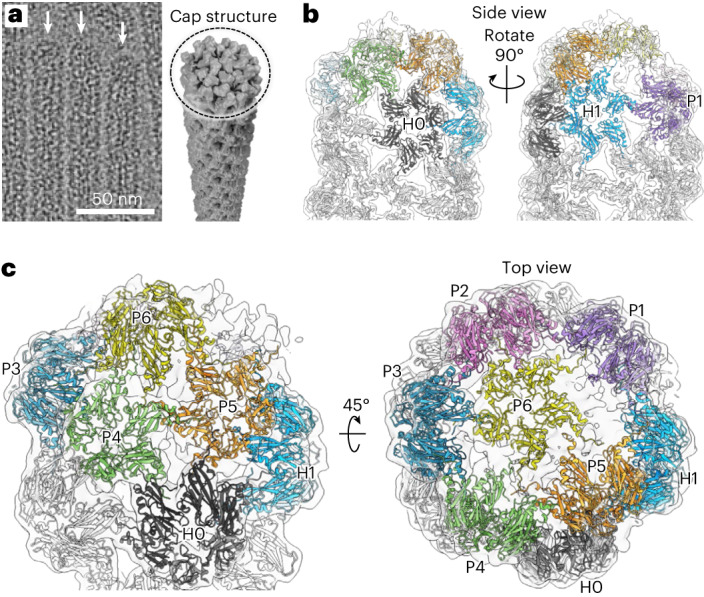
Single-particle reconstruction of the cap structure. **a**, Cryo-EM image of 6HB-2k tubes with caps indicated by white arrows (left) and schematic model of the cap structure (right). **b**,**c**, Reconstruction of the cap structure of the first CP layer with fitted models for CP hexamers (H0–H1) and pentamers (P1–P6). **b**, Side view. **c**, An oblique view (left) and top view (right) of the cap structure.

To confirm the encapsulation of DNA and for detailed structural characterization, single-particle reconstruction was performed based on cryo-electron microscopy (cryo-EM) (Fig. 2a,d and Supplementary Notes 5 and 6). Two-dimensional (2D) classification of particles picked along the filaments (Fig. 2b,e) and 3D refinement of ab initio 3D models, obtained from the recorded images only, results in helical structures with 4.3 Å resolution (Fig. 2c, left, and Supplementary Fig. 6a) for the first protein layer and 8 Å resolution (Fig. 2f and Supplementary Fig. 6c,d) for the second protein layer. The protein tubes are assembled of hexamers (Fig. 2c, top right), similar to tubes formed by in vitro assembled human immunodeficiency virus (HIV)^12^. For the atomic model (Fig. 2c, bottom right), CCMV CPs (PDB:1cwp) were flexibly fitted to the cryo-EM density maps.

The density in the lumen of the protein tubes corresponds to the encapsulated DNA origami (Fig. 2g). Its negative charge is evenly distributed (Fig. 2h, left). N-termini facing the origami create a positive electrostatic potential surface for the inner surface of the protein tube (Fig. 2h, bottom right). For the outer tube surface, in contrast, the negative electrostatic potential is predominant (Fig. 2h, top right, and Supplementary Fig. 7d), hence allowing the formation of protein multilayer complexes. We were not able to detect a specific physical contact point between the protein layers, or between DNA and protein, suggesting that unspecific electrostatic and protein–protein interactions were the driving force for the assembly.

Analysis of the protein layers shows a clear difference in the helical symmetry (Fig. 2i). Whereas the first layer is characterized by a 1-start helix with a turn of 63.8° and a rise of 15.4 Å (Supplementary Fig. 7a), the second layer has a 3-start helical symmetry with a turn of 38.8° and a 29.6 Å rise (Supplementary Fig. 7c,d).

Apart from the increased diameter of the tubular, complexed structures, the helical symmetry is in line with the properties of empty CCMV tubes observed by Bancroft et al.^18^. CCMV is known to adopt two distinct conformational states depending on the pH of the surrounding solution^18^. Upon increase of the pH from acidic to neutral and in the absence of metal ions, the capsid transforms from the native state into a swollen state. During this transformation, the morphology of both hexameric and pentameric units stays unchanged; however, the distance between the units increases by ~5 Å, most probably due to electrostatic repulsion^17^. The cryo-EM densities of CPs encapsulating 6HB (Fig. 2 and Supplementary Fig. 7) clearly show large holes between the hexameric units, resembling the swollen state, which is expected according to the solution conditions used.

The cap that closes the first CP layer around the origami (Fig. 3 and Supplementary Note 7) is found to consist of six pentamers and one hexamer, which create its curvature. Hexamer H0 (Fig. 3b,c, black) still follows the helical symmetry, whereas hexamer H1 (blue) is tilted inwards, most probably to be in contact with the pentamers. The pentamers (P1–P5) are arranged along the tubular axis and are responsible for creating the main curvature of the cap. Finally, the cap is sealed with a sixth pentamer (P6, yellow). Since the diameter of the first protein layer matches with *T* = 1 symmetry, and following from the helical geometry, six pentamers per tube end are expected to close the hexameric lattice, which is in line with our volume data.

## Evaluating the template versatility and protection

**Fig. 4:**
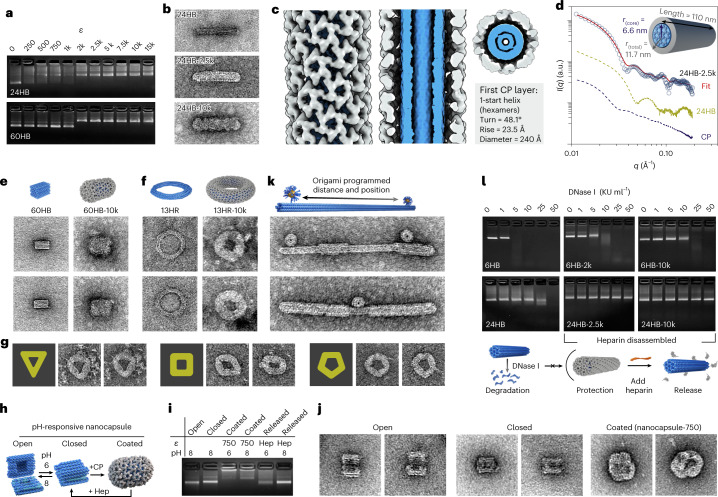
Applicability of capsid coating on structures with different thickness and shape. **a**, EMSA for 24HB and 60HB showing a decrease in the electrophoretic mobility with increasing *ε*. **b**, Negative-stain TEM images of plain 24HB and complexed structures with *ε* = 2.5k and *ε* = 10k. The image dimensions correspond to 200 nm × 100 nm. **c**, Cryo-EM density maps for 24HB-2.5k. The origami structure is highlighted in blue. **d**, SAXS scattering curves measured for CP, 24HB and 24HB-2.5k samples in solution. **e**, Negative-stain TEM images (125 nm × 125 nm) of plain 60HB and 60HB-10k. **f**, Negative-stain TEM images (125 nm × 125 nm) of 13HR structure before and after complexation with *ε* = 10k. **g**, Complexed 13HR (*ε* = 10k) can be classified into triangular (left), square-like (middle) and pentagonal (right) shapes. All images have dimensions of 125 nm × 125 nm. **h**,**i**, Schematic (**h**) and EMSA (**i**) of the different states the nanocapsule can adopt upon pH changes and coating. The coating is initially applied at pH 6 onto the closed nanocapsule, which can be removed by the addition of heparin (Hep). The pH can be changed either once coated or after decoating. **j**, Negative-stain TEM images of the plain nanocapsule when open, closed and coated (coating applied at pH 6, *ε* = 750). The image dimensions correspond to 100 nm × 100 nm. **k**, Application of protein coating on AuNP-functionalized 6HB structures. The image width corresponds to 400 nm. **l**, Stability of 6HB and 24HB upon DNase I treatment. For both structures, the digestion of the coated DNA origami (single (middle) or double protein layer (right)) is slower than for the plain structures (left). The coating has been removed by heparin before AGE to avoid retention in the wells. Source data

Our results show that 6HB is able to direct the assembly of CCMV CPs. Its diameter matches the size of the central channel of empty tubes^17,18,36^, and it simultaneously restricts the growth of long fibres. Furthermore, for other DNA origami structures tested (Fig. 4a for 24HB (top) and 60HB (bottom), and Supplementary Note 8 and Supplementary Fig. 9 for 13HR) the decrease in electrophoretic mobility confirms the existence of electrostatic interactions between the origami and CPs. Interestingly, for 60HB, complexation with CPs reveals two distinct states, whereas the tubular (6HB, 24HB) origami structures show a more gradual electrophoretic mobility shift in the gel upon complexation. It is notable that the shift occurs at similar *ε* values, independent of the origami structure, which is in line with a ‘magic ratio’ reported for the encapsulation of ssRNA^22^. The complete formation of a single CP layer on 24HB is observed at *ε* = 2.5k (Fig. 4b, middle, and Supplementary Fig. 5c), whereas a second CP layer requires *ε* = 10k (Fig. 4b, bottom, and Supplementary Fig. 5d). Compared to complexed 6HB, the diameter of complexed 24HB is slightly increased (21.9 ± 1.6 nm versus 18.1 ± 0.9 nm) and the helical symmetry (24HB-2.5k, single layer; Fig. 4c and Supplementary Figs. 6b and 7b) of the hexamers arranged in a 1-start helix differs (48.1° turn, 23.5 Å rise). Note that, in the cross section the highlighted 24HB origami structure (blue) is represented by two DNA layers.

The homogeneity of the complexed 24HB structures was confirmed by SAXS (Fig. 4d and Supplementary Note 9). This shows distinct curves for CPs only (dark blue) and 24HB (green), while a clear change in patterns is observed for complexed 24HB (24HB-2.5k, blue circles). A core–shell cylinder is chosen to represent the complexed structure best in a geometric model (red). The obtained radius of 6.6 nm for the core and a shell thickness of 5.1 nm results in a total *d*_SAXS_ = 23.4 nm, which is in agreement with the TEM analysis and the single-particle reconstruction of 24HB-2.5k.

Due to the low aspect ratio of 60HB (Fig. 4e) the formation of spherical shells rather than tubes might be expected because 60HB’s shorter edge corresponds to the outer diameter of the observed tubes. The coating of 60HB is found to develop from the square-shaped face of the origami structure, but fully complexed structures are only observed at large *ε*, with a clear trend toward smoothening of the edges of the complexes (Supplementary Note 10 and Supplementary Fig. 11a,b). A fully developed single-layer coating for 13HR similarly requires large *ε* (Fig. 4f and Supplementary Fig. 11c,d) although its cross-sectional size is between that of 6HB and 24HB, suggesting that the negative curvature is not well tolerated by the CPs. Following the tube formation along the template, clear kinks are seen, resulting in an appearance of the complexed structures predominantly as triangles, squares and pentagons (Fig. 4g). This implies that a series of short linear stretches deforming the template is more favourable for the assembly formation than a negative curvature.

Having established a system that allows precise control over the dimensions of the assembled structures (Supplementary Note 11 and Supplementary Fig. 12i), we aimed to further exploit the properties of DNA origami toward a more functional system. The nanocapsule, a dynamic, stimuli-responsive DNA origami, which can change its configuration from open to closed by decreasing the pH of the surrounding solution from 8 to 6 (ref. ^39^) (Fig. 4h–j) results in coated structures at *ε* ≥ 750 (Fig. 4i,j, Supplementary Note 12 and Supplementary Fig. 13c,d) at pH 6 (nanocapsule closed). An increase in pH after complexation leads to less aggregation, noticeable by an increase in the intensity of the leading band in the agarose gel, with a profile similar to observations when coating is performed at pH 8 (Supplementary Fig. 13a,b). The coating can be removed by the addition of heparin as a competitive agent, and adjusting the pH would return the nanocapsule to its open state. Additionally, since each strand of a DNA origami structure is addressable, functional units can be precisely positioned on the origami structure. We demonstrated this with DNA-coated gold nanoparticles (AuNPs) anchored on the 6HB structure (Fig. 4k, Supplementary Note 13 and Supplementary Fig. 14). As a result of the complexation, both the DNA origami and the AuNPs were coated, showing the formation of dumbbell-like structures with 6HB precisely controlling the distance between the spherical particles.

In addition, it is well known that DNA origami structures are susceptible to DNase I degradation^40^. Several strategies have been developed to circumvent the degradation, including the application of different coatings^41^. Complexation with CCMV CPs is found to enhance the stability of both 6HB and 24HB (Fig. 4l) until 50 Kunitz units (KU) ml^−1^ of DNase I (Supplementary Note 14 and Supplementary Fig. 15a). The complexed structures were disassembled after DNase I treatment with heparin (Supplementary Fig. 15b,c), exposing the plain structures to highly active DNase I for a short time, which is, however, long enough to attack the 6HB structure. The applied coating is highly efficient and has the ability to protect the structures also when incubated in cell medium supplemented with a final concentration of 5–10% fetal bovine serum (Supplementary Fig. 15d).

## Expanding the material toolbox

**Fig. 5:**
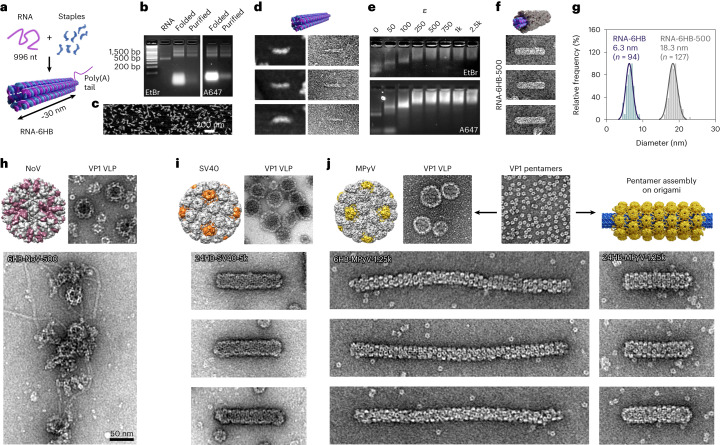
Variation of templating material and virus CPs. **a**, The RNA–DNA hybrid origami (RNA-6HB) is obtained by thermally annealing 996 nt RNA with DNA staples. The poly(A) tail was left unfolded. **b**,**c**, The successful folding can be monitored by AGE (**b**), in both the ethidium bromide (EtBr) and the Alexa647 (A647) channel, and by AFM (**c**). **d**, Folded structures selected from AFM (left) and negative-stain TEM (right) images of RNA-6HB. The image dimensions correspond to 125 nm × 75 nm. **e**, EMSA for RNA-6HB showing a decrease in the electrophoretic mobility with increasing *ε* in both the EtBr and the A647 channel. **f**, Negative-stain TEM images (125 nm × 75 nm) of complexed RNA-6HB structures with *ε* = 500. **g**, Observed size distributions (in diameter) for plain RNA-6HB (blue) and RNA-6HB-500 (grey). **h**, NoVLPs (top, TEM image 150 nm × 150 nm) are disassembled into VP1 units and assembled with 6HB at *ε* = 500 (bottom). **i**, VLPs built from the major CP, VP1, of SV40 (top, TEM image 150 nm × 150 nm) are disassembled into their pentameric subunits and reassembled onto 24HB at *ε* = 5k (bottom, TEM images 200 nm × 100 nm). **j**, Pentamers formed from the major CP, VP1, of MPyV (top middle, TEM image 150 nm × 150 nm) can either be assembled into VLPs (top left, TEM image 150 nm × 150 nm) or complexed with DNA origami (top right). Development of a pentamer-based protein layer on 6HB (bottom left, TEM images 400 nm × 100 nm) or 24HB (bottom right, TEM images 200 nm × 100 nm) at *ε* = 1.25k. Source data

The DNA origami templates presented here are all based on the common M13mp18 scaffold, but for some applications, DNA nanostructure functionality can be further increased through the rational design of their scaffold sequence. Recently, several RNA-based nanostructures, for example, RNA-scaffolded RNA–DNA hybrid origami structures, and their use in therapeutic applications have been reported^42–45^. Here, we have designed a 6HB RNA–DNA hybrid origami based on an RNA scaffold (RNA-6HB; Fig. 5a and Supplementary Note 15). Successful folding of the structure, including the integration of fluorophores (ATTO590), is suggested by AGE (Fig. 5b and Supplementary Fig. 16a) and by both atomic force microscopy (AFM) and TEM showing distinct structures with a length of *l*_avg_ = 40.3 ± 4.0 nm (Fig. 5c,d). Complexation with CPs results in decreased electrophoretic mobility (Fig. 5e) and a change in both length and diameter, from *d*_avg_ = 6.3 ± 1.0 nm for the plain structures to *d*_avg_ = 18.3 ± 1.2 nm for coated structures (*ε* = 500) (Fig. 5f,g and Supplementary Fig. 16b,c), similar to the increase observed for complexed 6HB DNA origami structures.

The versatility of our approach is evaluated by testing three different CPs, namely the major CPs, VP1s, of norovirus GII.4 (NoV; Fig. 5h, top), simian virus 40 (SV40; Fig. 5i, top) and murine polyoma virus (MPyV; Fig. 5j, top). Whereas NoV is known for its quasi-icosahedral *T* = 3 symmetry (180 VP1 copies)^46^, SV40 and MPyV adopt a *T* = 7d symmetry^47,48^. Notably, a polymorphic behaviour has been reported for all virus species used. For templating of NoV VP1 proteins, 6HB was mixed with virus-like particles (VLPs) and transferred into alkaline pH. However, the tested conditions did not show a notable electrophoretic mobility shift (Supplementary Fig. 17c). TEM reveals mainly plain 6HB origami, while origami templated protein structures could not be detected (Fig. 5h, bottom, and Supplementary Fig. 17d–f), most probably due to the lack of a positively charged N-terminal RNA binding domain on VP1 (Supplementary Note 16). In comparison, complexation of 24HB with SV40 capsomers, obtained after VLP disassembly, results in a highly ordered coating mainly consisting of pentameric subunits (24HB-SV40-5k; Fig. 5i, bottom). Packaging of nearly spherical DNA origami in SV40 VLPs^49^ and the assembly of VP1 into elongated structures in vitro has previously been reported^50–52^. In comparison to empty tubes, an increase in the tube diameter (*d*_avg_ = 26.3 ± 1.9 nm) is observed upon templating of the assembly on 24HB, with the tube length being partly heterogeneous (Supplementary Note 17). A similar, pentamer-based coating behaviour is observed for the second polyoma virus, MPyV (Supplementary Note 18). Polyoma viruses have not only been found to reassemble into pentamer-based tubule-like^53,54^ and spherical particles with both icosahedral and octahedral symmetry^53^ but also to interact with with foreign DNA^55^. Using DNA origami, the fully coated structures (*ε* ≥ 1.25k for both origami structures tested) display a narrow length and diameter distribution. The diameters of complexed 6HB and 24HB structures increase to *d*_avg_ = 21.5 ± 1.6 nm and *d*_avg_ = 27.2 ± 2.0 nm, respectively, while the lengths were found to be *l*_avg_ = 383.0 ± 12.3 nm and *l*_avg_ = 121.9 ± 3.1 nm.

## Conclusion

We have developed a strategy to direct the assembly of virus capsids at physiological conditions in a precise and programmable manner. By employing versatile DNA origami as templates, precise control over the size and shape of the formed assemblies can be achieved. The complexation is based on electrostatic interactions, which, due to the properties of the CPs, leads to the formation of one or two protein layers depending on the CP concentration used. It furthermore enables fine-tuning of the control over the assembly by altering the salt conditions or by engineering the CP–DNA interactions. Hexamers are the predominant building units of the CCMV CP-based protein layers, which were found to substantially differ in their symmetry depending on the template. Although protein coatings developed successfully on all tested origami shapes, a preference toward tubular origami structures was observed, hence allowing even the encapsulation of DNA origami with negative curvature. Moreover, we have demonstrated that the applicability of templating is neither limited to DNA origami but expandable to RNA-scaffolded origami, nor exclusive to CCMV CPs, resulting in assemblies built from other virus species.

Additionally, our approach does not only enhance the stability of DNA origami against nuclease digestion, but also enables the use of DNA origami as a highly functionalizable platform. The high addressability of DNA origami, which was demonstrated with AuNPs, can be leveraged in precise attachment of a variety of cargo/targeting molecules by hybridization or intercalation. In combination with functionalization of the protein components, the resulting multipurpose system could be implemented in various fields ranging from DNA-origami-based 3D display of ligands^56,57^ to gene delivery^58^.

## Methods

### DNA origami folding and purification

The DNA origami structures (6HB, 24HB, 60HB, 13HR and nanocapsule) were folded in a one-pot reaction by gradually decreasing the temperature using a Proflex 3 × 32-well PCR system (Thermo Fisher). The scaffold strands (p7249, p8064 and p7560 variants of single-stranded M13mp18) were purchased from Tilibit Nanosystems and the staple strands from Integrated DNA Technologies. To ensure high folding yields of DNA origami, structure-specific optimized conditions regarding both the annealing procedures and the buffer choice (‘folding buffer’, FOB) are used (Supplementary Note 20).

### Buffer exchange for DNA origami

The purified DNA origami structures were transferred into 6.5 mM 4-(2-hydroxyethyl)-1-piperazine ethanesulfonic acid (HEPES) buffer supplemented with 2 mM NaOH (HEPES-NaOH, pH 6.5) before complexation with CCMV CPs. The buffer exchange was performed by spin-filtration^59^ using 100 kDa molecular weight cut-off (MWCO) centrifugal filters (Amicon), which were washed before use by centrifuging with 400 μl of HEPES-NaOH buffer for 5 min at 14,000*g*. Subsequently, equal volumes of DNA origami solution and HEPES-NaOH buffer were added into the filter device and the centrifugation was continued for 10 min at 6,000*g*. A volume of HEPES-NaOH equal to 2.09× the initial volume of origami solution was then added, and the centrifugation step was repeated. The sample was collected by inverting the filter and centrifuging for 2.5 min at 1,000*g*.

### Isolation of CCMV CPs

The CPs were isolated from intact CCMV (for virus preparation, see Supplementary Note 21). Briefly, the virus particles were dialysed overnight against 50 mM Tris–HCl, 500 mM CaCl_2_ buffer, pH 7.5 supplemented with 1 mM dithiothreitol (DTT) using Slize-A-Lyzer Mini Dialysis cups (3.5 kDa MWCO, Thermo Scientific). The RNA was pelleted in a centrifugation step at 4 °C using 21,100*g* for 6 h, and the recovered supernatant was dialysed overnight against ‘clean buffer’ that contains 50 mM Tris–HCl, 150 mM NaCl at pH 7.5 supplemented with 1 mM DTT (adapted from ref. ^60^). The concentration of the proteins was determined based on their absorbance at 280 nm (extinction coefficient, 23,590 M^−1^ cm^−1^) using a BioTek Eon Microplate Spectrophotometer (2 μl sample, Take3 plate).

### AGE

AGE was used to study the binding interaction between the proteins and the origami structures by monitoring the shift in electrophoretic mobility. Furthermore, the intactness of the origami structures after folding and purification, and during DNase I digestion, was analysed by gel electrophoresis. To this end, samples (volumes ranging from 10 to 32 μl) supplemented with 6× gel loading dye (40% sucrose without dye for samples from digestion studies) were run in a 2% (w/v) agarose gel (1 × Tris–acetate–ethylenediaminetetraacetic acid (TAE) buffer, 11 mM MgCl_2_) for 45 min at 90 V in 1 × TAE buffer supplemented with 11 mM MgCl_2_. For staining, ethidium bromide (EtBr) at a final concentration of 0.46 μg ml^−1^ was used and the DNA was visualized under ultraviolet light using a GelDoc XR+ system (Bio-Rad).

### Complexation of DNA origami and CCMV CPs

The complexation between CPs and DNA origami was performed at a final origami concentration of 4 nM (10 μl samples). The origami was added in a 1:1 volume ratio to the protein solution that had been diluted in the ‘clean buffer’. Depending on the required protein excess, *ε*, which describes the molar ratio between CP to DNA origami, protein solutions ranging from 0 to 60 μM (corresponding to *ε* = 0–15k) were prepared. The NaCl concentration was adjusted to 150 mM, resulting in a complexation buffer containing 3.25 mM HEPES-NaOH, 25 mM Tris–HCl, 150 mM NaCl and 0.5 mM DTT. The complexation was performed at 4 °C for at least 1 h and subsequently analysed using AGE and TEM.

### DNase I digestion assays

To study the protection effect of the CP coating against degradation of the origami structures by DNase I, 2 μl of DNase I stock (ranging from 0 to 500 KU ml^−1^) was added to 16 μl of the sample. Additionally, CaCl_2_ and MgCl_2_ concentrations were adjusted, resulting in a final reaction volume of 20 μl containing 3.2 nM DNA origami, 2.6 mM HEPES-NaOH, 20 mM Tris–HCl, 120 mM NaCl, 0.4 mM DTT, 1 mM CaCl_2_ and 5 mM MgCl_2_. The samples are incubated at 37 °C for 15 min (6HB) and 60 min (24HB). Before analysing the outcome by AGE, samples complexed with CPs were disassembled using heparin sodium salt as a competitive binding agent (final concentrations of 1.5 μM for 6HB-2k and 24HB-2.5k and 82 μM for 6HB-10k and 24HB-10k; Supplementary Note 14).

### RNA–DNA origami folding and purification

For the RNA–DNA hybrid origami (RNA-6HB), EGFP mRNA (CleanCap EGFP mRNA, TriLink Bio Technologies, L-7601) was used as the scaffold. In a one-pot reaction, the 996-nt-long mRNA scaffold was thermally annealed with 29 staple strands (purchased from Integrated DNA Technologies, see Supplementary Note 22) into a short 6HB structure using a Proflex 3 × 32-well PCR system (Thermo Fisher). The structure is designed to contain two scaffold crossovers and has a helical pitch of 11 bp per turn. For the folding reaction, the mRNA and the staples were diluted into 1 × FOB containing 1 × TAE pH 8.4, 5 mM MgCl_2_ and 1 mM NaCl reaching final concentrations of 50 nM and 500 nM, respectively. The reaction mixture was incubated at 55 °C for 15 min^61^ and cooled down by placing it on ice for at least 10 min before storage at 4 °C. To validate the folding, four staple strands were exchanged with staple strands containing a 3′ overhang (labelled with F, Supplementary Table 2). A fluorophore-containing attachment strand (ATTO590, Integrated DNA Technologies), which was added to the folding mixture in 10× excess per attachment site, can then be integrated into the structure by hybridization with the staple overhangs.

The folded structures were purified from excess staple strands by spin-filtration. To this end, the filter (100 kDa MWCO, Amicon) was washed with 400 μl of 1 × FOB by centrifugation at 14,000*g* for 5 min, followed by two-times addition of 40 μl RNA-6HB together with 40 μl of 1 × FOB. After a centrifugation step at 6,000*g* for 10 min, 80 μl of 1 × FOB was added and the centrifugation continued (6,000*g*, 10 min). This washing step was repeated in total three times before the sample was recovered by inverting the filter into a clean tube (1,000*g*, 2.5 min). The concentration was determined by measuring the absorbance at 260 nm (extinction coefficient, 1.29 × 10^7^ M^−1^ cm^−1^), and the successful folding was determined by AGE (3.5 % (w/v) gels, visualization under ultraviolet light (EtBr channel) and red light (A647 channel), ChemiDoc MP system, Bio-Rad), AFM and TEM.

### Complexation of RNA-6HB origami and CCMV CPs

For the complexation, purified RNA-6HB origami in 1 × FOB was mixed with CCMV capsids in ‘clean buffer’ in a 1:1 ratio at a final hybrid origami concentration of 7.5 nM. This results in a complexation buffer containing 45 mM Tris, 75.5 mM NaCl, 10 mM acetic acid, 2.5 mM MgCl_2_, 0.5 mM DTT and 0.5 mM EDTA. The samples were incubated at 4 °C for at least 1 h before analysis with AGE and TEM.

### Complexation of DNA origami and NoV CPs

NoVLPs were prepared as reported by Lampinen et al.^62^ and stored in 1 × phosphate-buffered saline (PBS, 137 mM NaCl, 2.7 mM KCl, 10 mM Na_2_HPO_4_ and 1.8 mM KH_2_PO_4_, pH 7.4); however, here SpyTag003 (ref. ^63^) has been fused to the C-terminus of the VP1 from the NoV strain Hu/GII.4/Sydney/NSW0514/2012/AU. The particles were quality controlled with dynamic light scattering for particle formation, sodium dodecyl sulfate polyacrylamide gel electrophoresis for protein purity and the residual dsDNA was measured. For the complexation with DNA origami, DNA origami was present in the sample during both disassembly and reassembly of the VLPs. To this end, the origami structures were transferred into deionized water using spin-filtration (as described above). The DNA origami was mixed with the NoVLPs at different concentrations in a 1:4 (v/v) ratio, resulting in a final origami concentration of 6 nM (30 μl samples). The samples were transferred into 3.5 kDa MWCO dialysis cups (Slize-A-Lyzer, Thermo Scientific) and dialysed overnight at 4 °C against 50 mM Tris–HCl, pH 8.9. For reassembly, the samples were, in a second step, dialysed overnight at 4 °C against 100 mM sodium phosphate buffer, pH 6.0, similarly as reported by White et al.^64^ The complexation during disassembly and assembly of the NoVLPs was analysed by AGE and TEM.

### Complexation of DNA origami and SV40 CPs

The SV40 major CP VP1 (abcam, ab74565) was disassembled and reassembled (adapted from ref. ^50^) by dialysing the assembled VLPs in PBS against 20 mM Tris, 2 mM DTT, 5 mM EDTA and 50 mM NaCl, pH 8.9 for 2 h at 4 °C (3.5 kDa MWCO, Slize-A-Lyzer, Thermo Scientific), after which the EDTA concentration was decreased by an additional dialysis step at 4 °C for 2 h against 20 mM Tris, 2 mM DTT, 2 mM EDTA and 50 mM NaCl, pH 8.9. The concentration was determined based on the absorbance at 280 nm (VP1 extinction coefficient, 32,890 M^−1^ cm^−1^). The DNA origami was transferred into 100 mM HEPES buffer, pH 7.2, supplemented with 125 mM NaCl by spin-filtration (as described above). The proteins were mixed with the DNA origami in 1:1 (v/v) ratio to reach final concentrations of 0–20 μM and 2 nM, respectively, and the samples were incubated for 24 h at room temperature before analysis using AGE and TEM.

### Complexation of DNA origami and MPyV CPs

For the complexation of VP1 capsomers (for recombinant expression and purification, see Supplementary Note 23) and DNA origami, the origami structures were first transferred into 40 mM Tris buffer, pH 8.0, supplemented with 20 mM acetic acid, 2 mM EDTA and 12 mM MgCl_2_ using spin-filtration (see above). Depending on the desired excess of proteins, *ε*, the capsomers were diluted into ‘storage buffer’, containing 40 mM Tris, 200 mM NaCl, 1 mM EDTA, 5% (v/v) glycerol and 5 mM DTT, pH 8.0. For the complexation, VP1 capsomers were diluted in a ratio of 1:20 in the origami solution, resulting in a final origami concentration of 0.75 nM (30 μl samples) and a complexation buffer containing 40 mM Tris, 19 mM acetic acid, 1.95 mM EDTA, 11.4 mM MgCl_2_, 10 mM NaCl, 0.25% (v/v) glycerol and 0.25 mM DTT, pH 8. The complexation reaction was incubated at 4 °C overnight before analysis with AGE and TEM.

### AFM

A 20 μl droplet of 10 nM RNA-6HB origami solution (MgCl_2_ concentration adjusted to 12.5 mM) was deposited on a freshly cleaved mica substrate (Electron Microscopy Sciences) for 1 min, followed by three washing steps with 100 μl deionized water that was immediately blotted away. The sample was dried under a steady nitrogen stream and imaged immediately after sample preparation. AFM images were acquired in air using ScanAsyst in Air Mode together with ScanAsyst-Air probes (Bruker) on a Dimension Icon AFM (Bruker). Image processing was performed in NanoScope Analysis v.1.90 (Bruker).

### TEM

Plain DNA origami samples (4 nM) were prepared by incubation of a 3 μl droplet for 3 min on a plasma cleaned (20 s oxygen plasma flash, Gatan Solarus) Formvar carbon-coated copper grid (FCF400Cu, Electron Microscopy Sciences), which was subsequently blotted against filter paper and negative stained. For CCMV-CP-complexed samples (4 nM DNA origami), a 3 μl droplet was deposited on the grid for 1.5 min. After blotting against filter paper, the grid was immersed in a 10 μl droplet of complexation buffer (3.25 mM HEPES-NaOH, 25 mM Tris–HCl, 150 mM NaCl, 0.5 mM DTT) for 5 s. For samples with DNA origami concentrations ≤2 nM (for example, complexation with SV40, MPyV), and for samples containing RNA-6HB (7.5 nM origami concentration), the droplet size was increased to 5 μl and the incubation time extended to 5 min. Negative staining^65^ was performed by first immersing the grid in a 5 μl droplet of aqueous 2% (w/v) uranyl formate solution (supplemented with 25 mM NaOH for pH adjustment), which was immediately blotted away. This step was followed by an immersion in a 20 μl droplet, which was incubated on the grid for 45 s. After the final blotting step, the samples were left to dry for at least 20 min before imaging was performed on a FEI Tecnai 12 Bio-Twin microscope at an acceleration voltage of 120 V.

### Cryo-EM

The samples for cryo-EM were prepared using a vitrification apparatus (Vitrobot, Thermo Fisher Scientific). The origami concentrations in the complexed samples were 90 nM for 6HB-2k, 84 nM for 24HB-2.5k, 18 nM for 6HB-10k and 21 nM for 24HB-10k, resulting in total CP concentrations of 180 μM and 210 μM for complexed 6HB and 24HB samples, respectively. A 3 μl aliquot of the complexed origami sample was deposited on a plasma-cleaned (50 s, Harrick Plasma PDC-002-EC instrument) holey carbon-coated grid (copper 200 mesh R1.2/1.3, Quantifoil). After a 1 min incubation, excess liquid was blotted for 10 s at 100% relative humidity and 6 °C, followed by plunging the grid into liquid ethane. The grids were stored in liquid nitrogen. Data were collected at liquid nitrogen temperature in a Talos Arctica transmission electron microscope (Thermo Fisher Scientific) operated at 200 kV, using a Falcon III direct electron detector (Thermo Fisher Scientific). A magnification of 150,000× was used, resulting in a calibrated pixel size of 0.96 Å. The data collection parameters are listed in Supplementary Table 4 (Supplementary Note 24).

### Single-particle reconstruction

Cryo-EM data were processed using CryoSPARC 3.3.2 (Structura Biotechnology) unless stated otherwise. Contrast transfer function parameters were estimated using CTFFIND4 (ref. ^66^). Segments along filaments were defined using the Filament Tracer function. Helical symmetry parameters were estimated initially from 2D class averages using Python-based Helix Indexer^67^. The structure and helical symmetry parameters were refined using Helix Refine function and non-uniform refinement on motion-corrected helix segments. To determine the helical symmetry parameters of the 6HB-10k outer layer, a second 2D classification run was performed after subtracting the contribution of the inner layer using the Particle Subtraction function. The Helix Refine was run on the subset of particles that showed a clear second layer, using the determined symmetry parameters as initial estimates. Reconstructions were sharpened by applying an ad hoc *B*-factor of −300 Å^2^. The reconstructions were averaged in real space by imposing the helical symmetry parameters on the central, most ordered part of the map (50% of the volume) in Bsoft^68^.

For modelling the structure of the capsomer, CP monomer (PDB:1cwp) was fitted in the 6HB-2k reconstruction in the six positions of the hexamer as rigid bodies in UCSF ChimeraX 1.3 (ref. ^69^). The atomic model was refined against the density using ISOLDE 1.3 (ref. ^70^) and Phenix 1.19 (ref. ^71^). To create atomic representations of the filaments, symmetry copies of the hexamer were created in ChimeraX. To visualize the placement of CP hexamers and pentamers in the cap, the caps of the 6HB-2k filament were manually picked in the micrographs. The cap structure was refined using the Helix Refine function omitting symmetrization, as this allowed limiting the tilt angle of the caps close to side views. Reconstruction of the cap was filtered to its local resolution using Local Filter. The hexamer atomic model and previously determined pentamer structure (extracted from PDB:1cwp after applying icosahedral symmetry) were fitted as rigid bodies in ChimeraX 1.3. Data-processing parameters are given in Supplementary Table 4. Model refinement and validation parameters are shown in Supplementary Table 5.

### SAXS

The samples for SAXS were prepared at origami concentrations of 165 nM (6HB, corresponding to a disassembled CP concentration of 330 μM) and 180 nM (24HB, corresponding to a disassembled CP concentration of 450 μM) and sealed within a 1.5-mm-diameter glass capillary. The measurements were performed using a Xenocs Xeuss 3.0C device equipped with a GeniX 3D copper microfocus source (wavelength *λ* = 1.542 Å) and an EIGER2 R 1M hybrid pixel detector at a sample–detector distance of 1,100 mm. Data acquisition was performed for 3 × 3 h per sample. To obtain the 1D SAXS data, the 2D scattering data were azimuthally averaged. The magnitude of the scattering vector *q* is given by $q\,=\,4\uppi \sin \theta /\lambda$ with 2*θ* being the scattering angle. Data treatment included averaging of the triplicate 2D data of each sample, background subtraction from the complexation buffer (3.25 mM HEPES-NaOH, 25 mM Tris–HCl, 150 mM NaCl, 0.5 mM DTT) and a form factor was fitted to a cylinder (6HB, 24HB), spheres (*T* = 3 icosahedral CPs assemblies) and a core–shell cylinder (6HB-2k, 24HB-2.5k) using SasView software. A Debye–Anderson–Brumberger model was added to account for the background.

## Online content

Any methods, additional references, Nature Portfolio reporting summaries, source data, extended data, supplementary information, acknowledgements, peer review information; details of author contributions and competing interests; and statements of data and code availability are available at 10.1038/s41565-023-01443-x.

### Supplementary information

Supplementary Information Supplementary information for publication. Supplementary Notes 1–24, Figs. 1–20 and Tables 1–5.

### Source data

Source Data Fig. 1 Measurement data (diameter) from TEM.

Source Data Fig. 4 Plot data for SAXS.

Source Data Fig. 5 Measurement data (diameter) from TEM.

## Data Availability

All data generated or analysed during this study are included in this published article and its Supplementary Information files (including Supplementary Notes 1–24, Supplementary Figs. 1–20 and Supplementary Tables 1–5) or are available from the corresponding authors upon request. Cryo-EM maps and atomic models reported in this study have been deposited in the Electron Microscopy Data Bank (EMDB) under accession codes EMD-16076, EMD-16077, EMD-16078, EMD-16079, EMD-16080 and in the Protein Data Bank (PDB) under accession identifier PDB 8BI4. Source data are provided with this paper.
