# Supplementary material for: DNA-origami-directed virus capsid polymorphism
Source: Nat Nanotechnol. 2023 Jul 17;18(10):1205–12. doi: 10.1038/s41565-023-01443-x (PMC10575778; doi:10.1038/s41565-023-01443-x)
Supplement: Supplementary file 1 — Supplementary information for publication. Supplementary Notes 1–24, Figs. 1–20 and Tables 1–5. [file 41565_2023_1443_MOESM1_ESM.pdf]

nature nanotechnology

Article https://doi.org/10.1038/s41565-023-01443-x

DNA-origami-directed virus capsid polymorphism

In the format provided by the authors and unedited

Contents

	Page
Note S1: Folding of DNA origami structures (Supplementary Figure 1)	S2
Note S2: SAXS analysis of complexed 6HB (Supplementary Figure 2)	S3
Note S3: Quantification of the coating yield (Supplementary Figure 3)	S4
Note S4: Growth of first CP layer (Supplementary Figure 4)	S6
Note S5: Supplementary cryo-EM micrographs (Supplementary Figure 5)	S7
Note S6: Single-particle reconstruction (Supplementary Figures 6–7)	S8
Note S7: Reconstruction of the cap structure (Supplementary Figure 8)	S10
Note S8: Coating of 13HR structure (Supplementary Figure 9)	S11
Note S9: SAXS analysis of complexed 24HB (Supplementary Figure 10)	S12
Note S10: Negative-stain TEM images (Supplementary Figure 11)	S13
Note S11: Dimensions of coated structures (Supplementary Figure 12)	S14
Note S12: Coating of the nanocapsule structure (Supplementary Figure 13)	S15
Note S13: Functionalization of 6HB (Supplementary Figure 14)	S16
Note S14: DNase I digestion studies (Supplementary Figure 15)	S17
Note S15: RNA-DNA hybrid origami (Supplementary Figure 16)	S18
Note S16: Coating with norovirus (NoV) (Supplementary Figure 17)	S19
Note S17: Coating with simian virus 40 (SV40) (Supplementary Figure 18)	S20
Note S18: Coating with murine polyoma virus (MPyV) (Supplementary Figure 19)	S21
Note S19: Materials	S22
Note S20: Folding and Purification of DNA origami (Supplementary Table 1)	S22
Note S21: Isolation of native CCMV	S23
Note S22: Staple list for RNA-DNA hybrid origami (Supplementary Table 2)	S24
Note S23: Recombinant expression and purification of MPyV capsomers (Supplementary Figure 23, Supplementary Table 3)	S25
Note S24: Collection of parameters used in cryo-EM and single-particle reconstruction (Supplementary Tables 4–5)	S27
Supplementary Information references	S28

Note S1: Folding of DNA origami structures

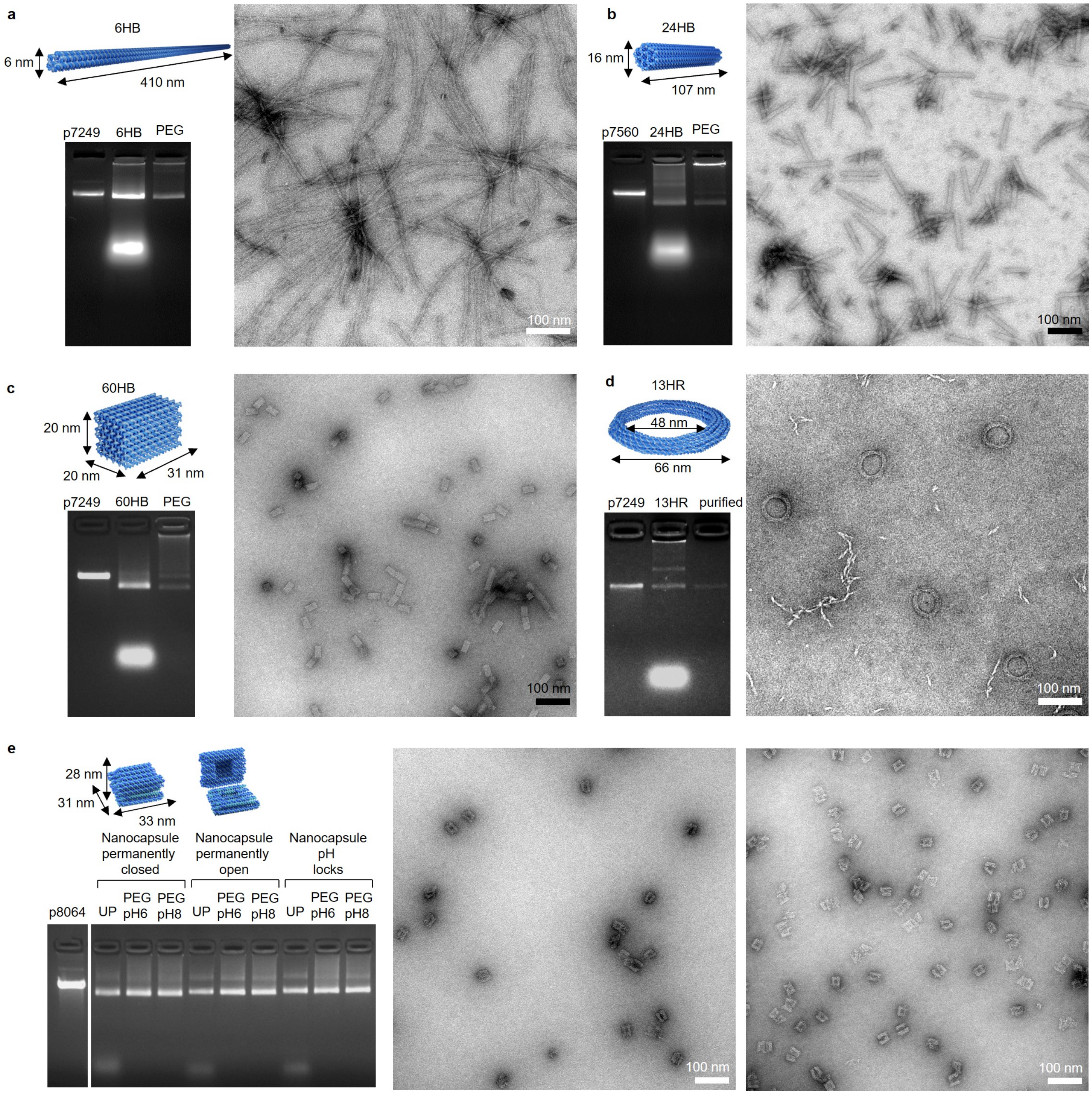

Fig. S1 Characterization of the DNA origami structures, **a**, 6HB, **b**, 24HB, **c**, 60HB and **d**, 13HR. For each structure, a schematic showing the dimensions (top left) and the corresponding TEM micrograph (right) are shown. Agarose gel electrophoresis (AGE, bottom left) shows the folded structures before (lane 2) and after purification (lane 3) in comparison to the scaffold (lane 1). **e**, For the nanocapsule the structures were transferred into buffers at both pH 6 and pH 8 after purification, which is especially important for the structures with the pH-responsive locks. AGE (bottom left) shows the folded nanocapsule variations before (UP) and after purification. Negative-stain TEM confirms the closed (middle) and open (right) conformations of the nanocapsule.

Note S2: SAXS analysis of complexed 6HB

The homogeneity of complexed structures in a 6HB-2k sample was evaluated by SAXS. For modelling purposes, the background in form of the complexation buffer was subtracted, simultaneously with the addition of a Debye background (Fig. S2a after and Fig. S2b before background treatment). For plain 6HB (green) and 6HB-2k (blue circles), clear features are displayed in the intensity distributions. 6HB-2k was geometrically modelled using a core-shell cylinder, resembling 6HB as the core cylinder and the single protein layer as its shell. A radius of 3.1 nm for the core ($r_{(\mathrm{core})}$) and 10.2 nm for the core-shell cylinder ($r_{(\mathrm{total})}$) are in agreement with the dimensions obtained from TEM analysis and cryo-TEM reconstruction (main article Fig. 2). It is notable that in comparison to 24HB (main article Fig. 4d), the radius of the shell is slightly increased for 6HB. Assuming a consistent thickness of the protein layer, and considering the thickness of 6HB, these results suggest a certain degree of freedom of 6HB within the protein shell. To account for the semi-solid intra-bundle distance between the DNA helices, a Lorentzian peak was included in the model (1). Its center is at 0.127 Å^{-1}, corresponding to an inter-helical diameter of 3.3 nm (Fig. S2c, orange dashed). The length of the structure was fixed to 4,000 Å, and has no impact on the features observed since it is outside of the experimental range.

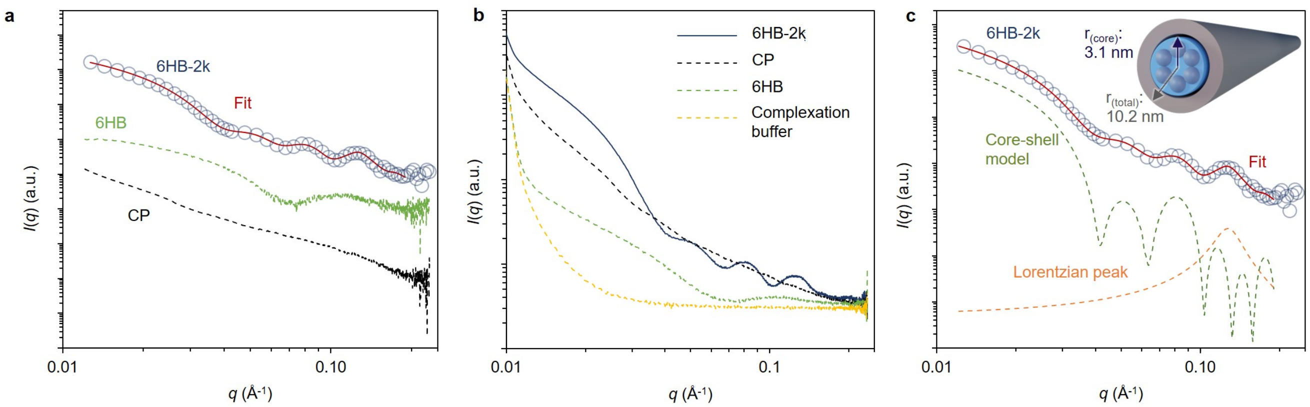

Fig. S2 a, SAXS scattering curves measured in solution for 6HB-2k, plain 6HB (green) and CPs only (blue). **b**, Intensity distributions of 6HB-2k (blue), CPs only (dark blue dashed), plain 6HB (green dashed) and the complexation buffer (yellow dashed) before buffer substraction. **c**, Breakdown of the fit for 6HB-2k to show the contributions of the chosen core-shell model (green dashed) and the Lorentzian peak (orange dashed).

Note S3: Quantification of the coating yield

Observed abnormalities when analyzing complexed samples at either ε = 2k or ε = 10k were collected into four defect classes, namely loops (Fig. S3a), bends (Fig. S3b), structures with incomplete layers (Fig. S3c,g), and elongated structures (Fig. S3d). Both bends and loops can either arise from a defect in the template, or by a T = 1-like defect upon CP assembly. Introduction of pentamers can create a local curvature, yielding in bending of the rather flexible 6HB template. A similar behaviour is observed for the CP assembly on 13HR (main article Fig. 4f,g), where it is more pronounced due to the constrained shape. The fraction of bend defects, φ, can be determined by

$$\varphi = \frac{\ell}{n \cdot \theta} \tag{S1}$$

where ℓ describes the measured tube length, n the number of observed bends, and θ the measured bend length. Using this equation, θ was calculated to be 0.46 % for 6HB-2k and 0.70 % for 6HB-10k.

The appearance of elongated structures seems to be concentration dependent. They were mainly observed in samples complexed at high final origami concentrations (c $\geq$ 80 nM), at which the denseness of the 6HB increases and therefore also the propensity of encapsulation of more than one single origami structure.

Most frequently, incomplete layers were observed, especially for structures complexed at ε < 2k and ε < 10k with regards to single (grey) and double (green) layer formation, respectively (Fig. S3e). The formation of double layer structures requires considerably greater ε than single layer structures, most likely due to the decreased strength of negative surface charge. Subsequently, a considerable part of free CPs assembles into T = 3 particles. When assembling into the second protein layer, the nucleation seems to happen nonspecifically along the entire structure (Fig. S3g, 6HB-7.5k). For batch characterization of free CPs, an agarose gel (Fig S3f, top), showing the decrease in electrophoretic mobility, was post-stained with Coomassie blue to visualize the proteins. Staining was performed for 1 h. After stain removal and three washing steps in water á 5 min, the gel was destained for 2 h in 40 % ethanol, 10 % acetic acid before imaging. At ε $\geq$ 2k, the protein concentration is high enough to be detected (Fig. S3f, bottom) and the protein bands can be compared to the DNA origami bands. While the leading band is overlapping in both EtBr and Coomassie channel, a second band develops, which is trapped in the wells and/or runs slightly into the opposite direction, supposedly free CPs.

Negative-stain TEM images of 6HB-1k (Fig. S3h) and 6HB-5k (Fig. S3i) illustrate the distribution between different fractions described in Fig. S3e.

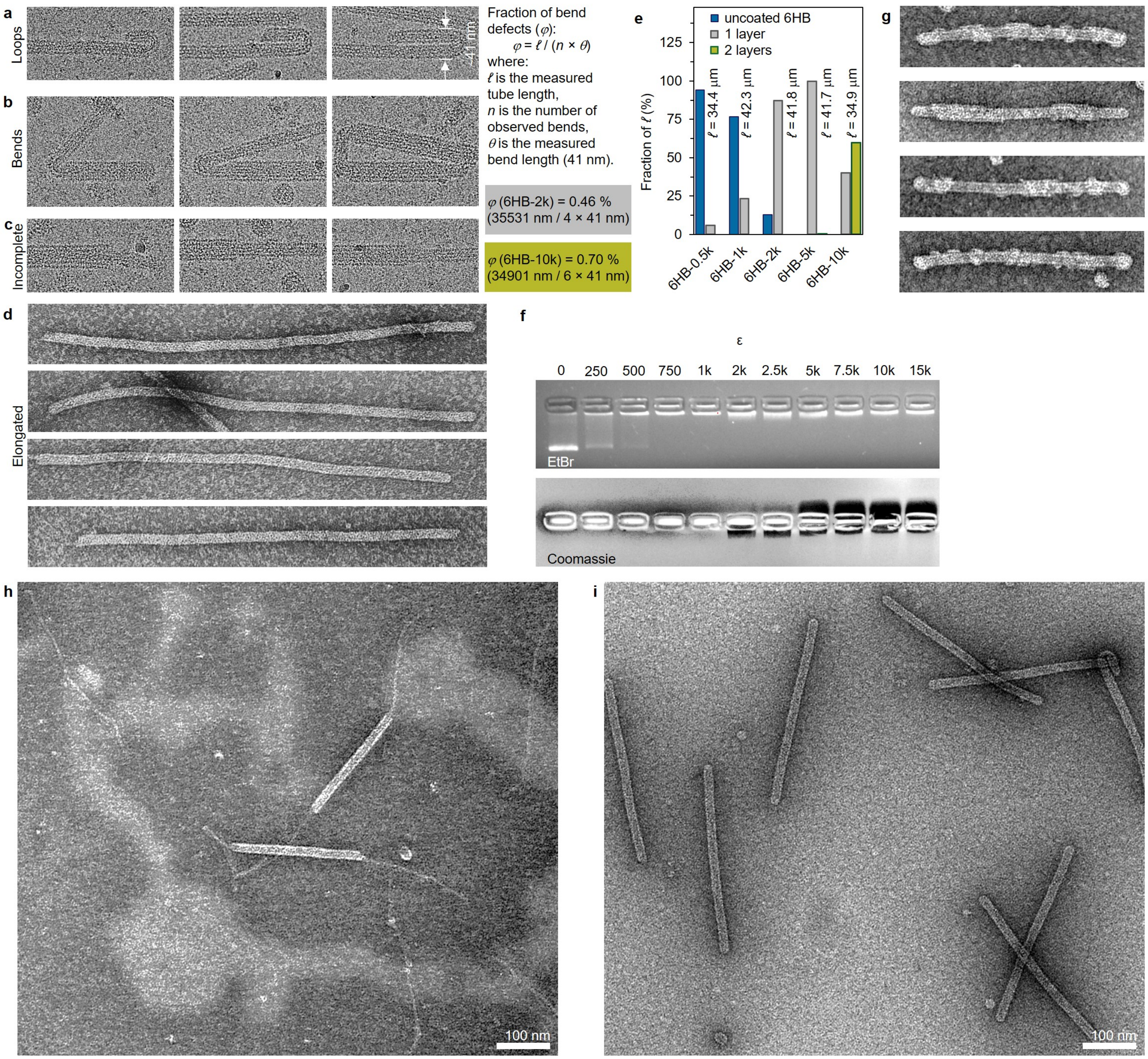

Fig. S3 Structural defects observed for complexed 6HB structures in negative-stain and cryo-TEM classified into **a**, loops (images 200 nm ×100 nm), **b**, bends (images 200 nm ×150 nm), **c**, incomplete layers (images 200 nm ×100 nm), and **d**, elongated structures (images 750 nm ×100 nm). **e**, Quantification of free origami (blue), and parts coated with single (grey) and double (green) layer in samples with varying ε. **f**, EMSA of 6HB following the shift of both the origami (EtBr channel, top) and proteins (Coomassie, bottom). **g**, Negative-stain TEM images showing the development of the second CP layer at ε = 7.5k (images 400 nm ×100 nm). Negative-stain TEM images of **h**, 6HB-1k and **i**, 6HB-5k.

Note S4: Growth of first CP layer

The dependency between the diameter of the rod and the preferred location of nucleation along the rod was suggested to be related to the ratio (γ) between the spontaneous curvature radius of the capsomers and the radius of the template. The spontaneous curvature radius refers thereby to the inner radius of the sphere the capsomers would assemble into (2). From a continiuum expression, derived by Lazaro *et al.* (2) for BMV a threshold (γ^*) can be predicted which separates the assembly preferentially on spherical (cap) from cylindrical surfaces. For $\gamma \gg \gamma^*$, capsomers were suggested to nucleate along the rod-like region whereas for $\gamma \ll \gamma^*$, nucleation on the spherical ends would be favoured. The two regions cross over around γ^* (2). Using the total diameters of both plain origami and complexed structures calculated from the SAXS measurements, $d_{total,6HB-2k}$ = 20.4 nm and $d_{total,24HB-2.5k}$ = 23.4 nm, and taking the size of the capsid protein, 3.8 nm (3), into consideration, the ratio could be calculated. Assuming a similar threshold as for BMV, $\gamma^* \sim 1.6$ (2), the CPs should preferentially nucleate along the origami for 6HB and from both the ends and along the origami for 24HB, which is also observed in TEM, as shown for 6HB-500 (Fig. S4a), 24HB-250 (Fig. S4b), and 24HB-750 (Fig. S4c).

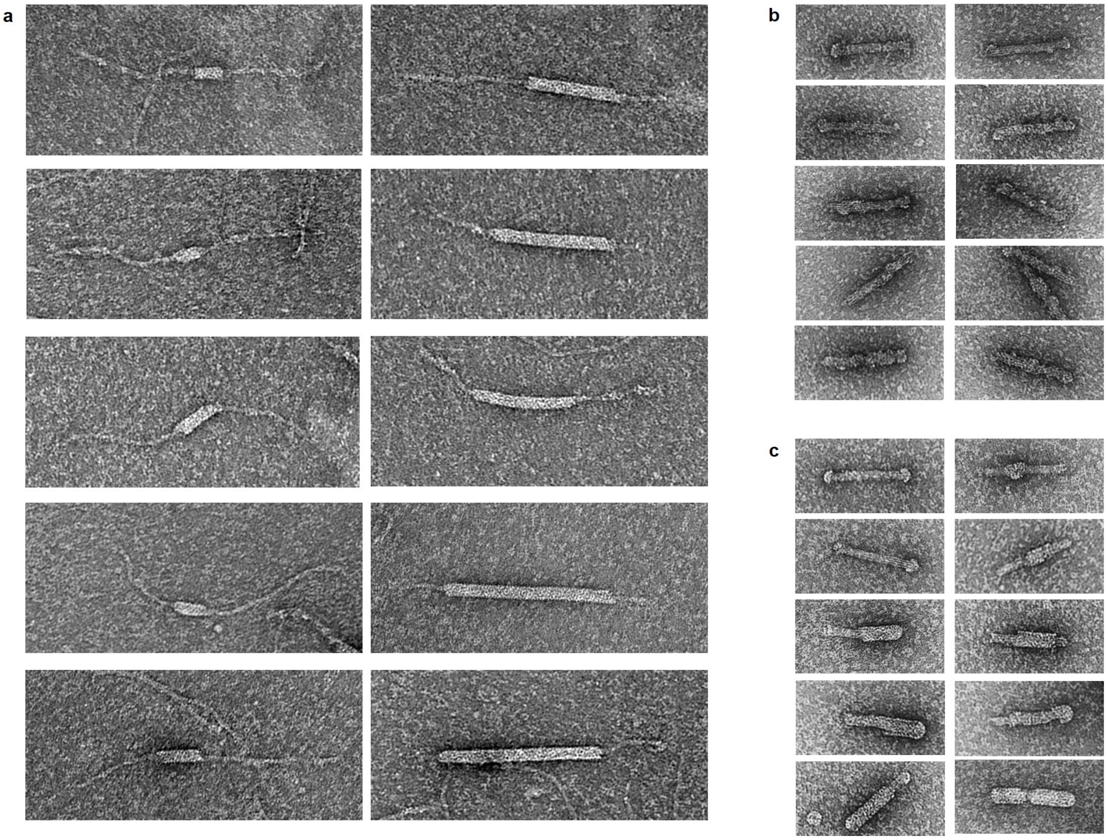

Fig. S4 **a**, 6HB-500, **b**, 24HB-250, and **c**, 24HB-750 showing the development of the first CP layer. The image dimensions correspond to 450 nm × 200 nm for **a**, and 150 nm × 100 nm for **b,c**.

Note S5: Supplementary cryo-EM micrographs

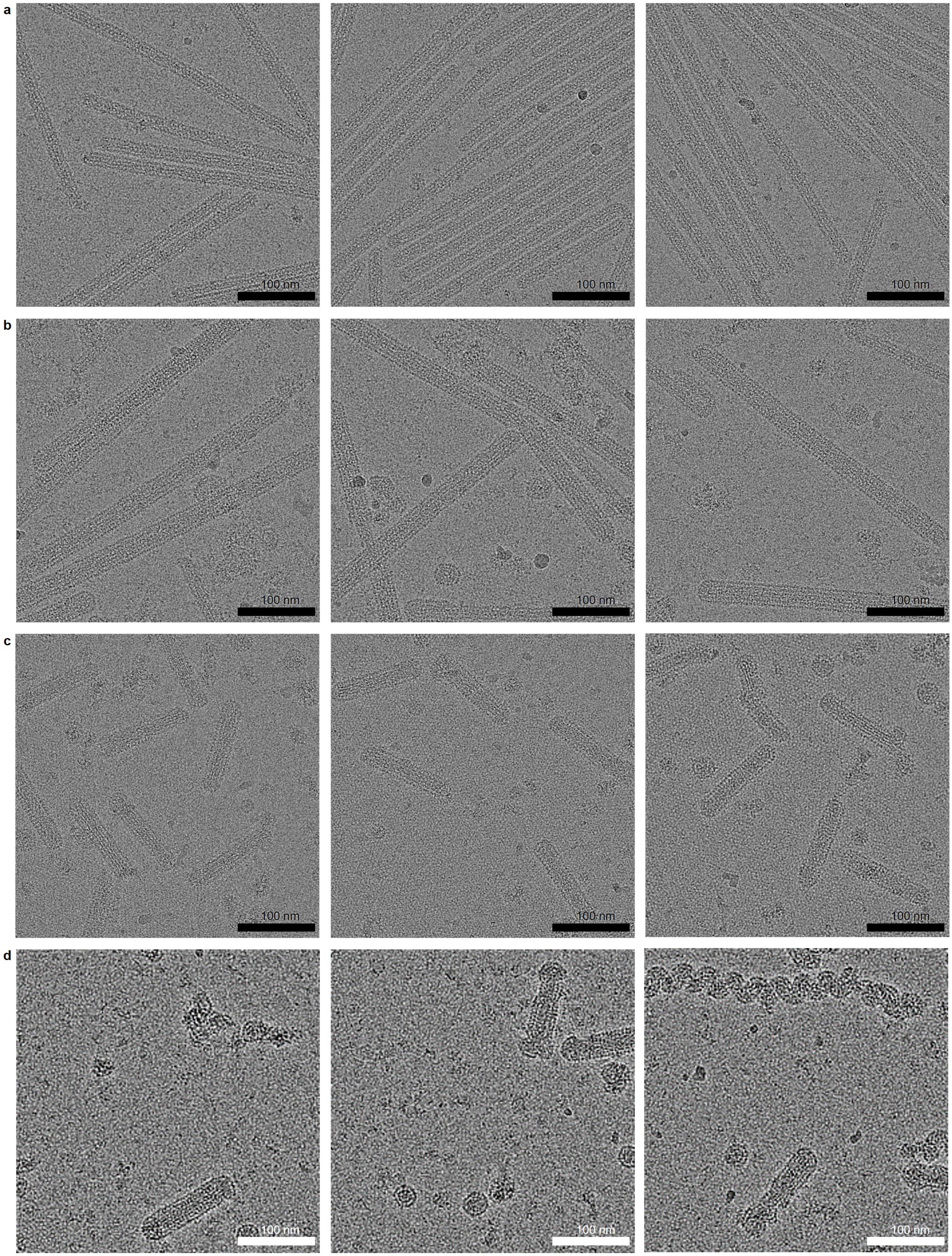

Fig. S5 Supplementary cryo-EM micrographs for **a**, 6HB-2k, **b**, 6HB-10k, **c**, 24HB-2.5k and **d**, 24HB-10k samples.

Note S6: Single-particle reconstruction

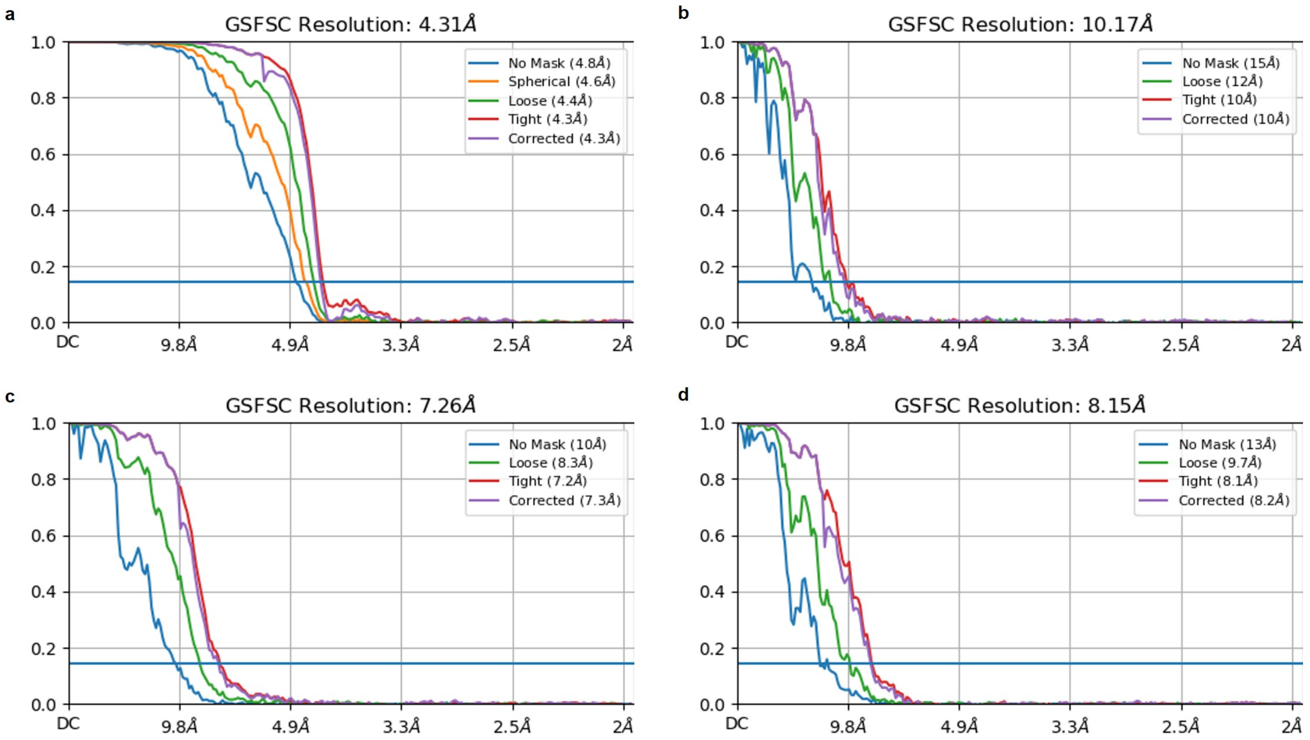

Fig. S6 Resolution estimates by "gold standard Fourier shell correlation" (GSFSC) for **a**, 6HB-2k, **b**, 24HB-2.5k, **c**, 6HB-10k, first CP layer and **d**, 6HB-10k, second CP layer.

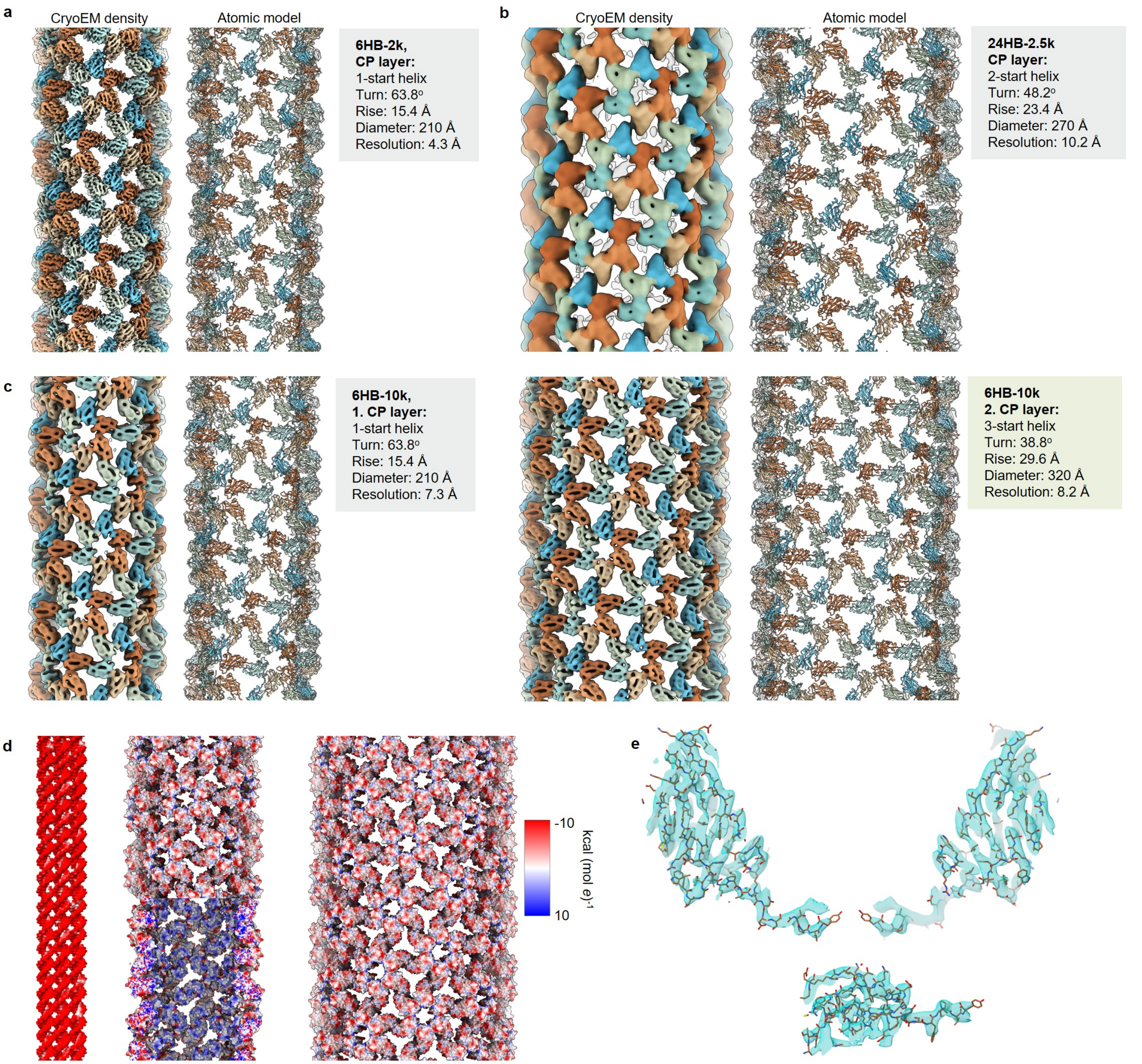

Fig. S7 Cryo-EM densities (left) and atomic models (right) and characteristics for the CP layers of **a**, 6HB-2k, **b**, 24HB-2.5k and **c**, 6HB-10k. **d**, Electrostatic potential surfaces for 6HB (left), the first CP layer (middle) and the second CP layer (right). **e**, Map to model comparison for the protein shell of 6HB-2k.

Note S7: Reconstruction of the cap structure

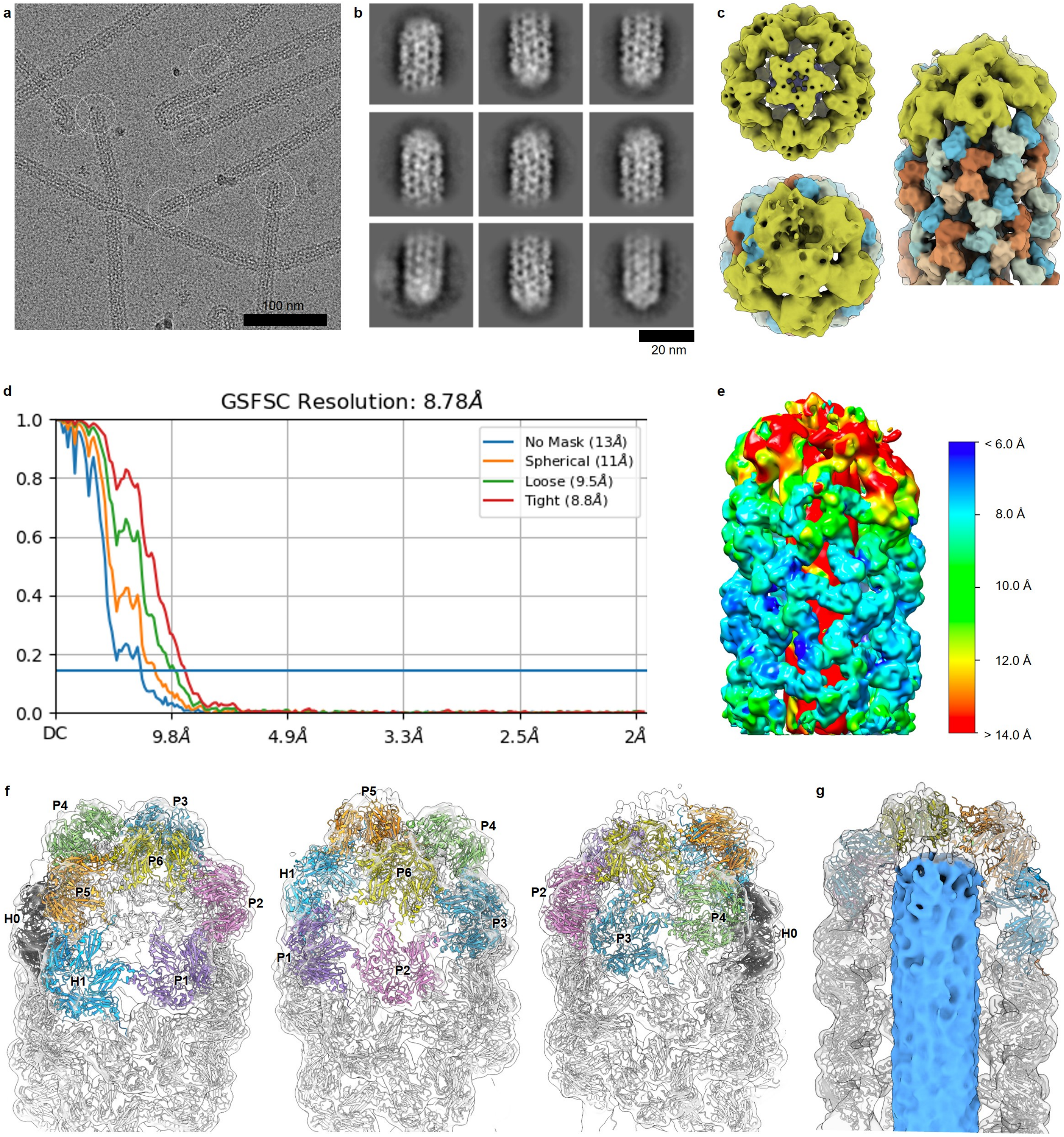

Fig. S8 a, Representative cryo-EM micrograph showing the selection of the filaments' ends. **b**, Selected 2D class averages. **c**, Comparison between an "empty" spherical particle with *T* = 1 symmetry (top left; calculated from the 6HB-2k data by icosahedral single particle reconstruction) and the cap structure of 6HB-2k top (bottom left) and side view (right). **d**, Resolution estimates by GSFSC. **e**, Local resolution of the cap structure. **f**, Supplementary views of the cap structure showing the positioning of the hexamers H0-1 (black, blue) and the pentamers P1-6. **g**, Cross-section showing the position of 6HB (blue) in the capped structure.

Note S8: Coating of 13HR structure

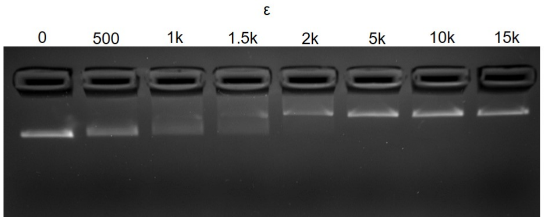

Fig. S9 The complexation between 13HR and CCMV CPs is monitored using AGE.

Note S9: SAXS analysis of complexed 24HB

For the model, the buffer (Fig. S10a) was subtracted, while a Debye background was added. Additionally, CPs assemble spontaneously into icosahedral structures (4). They can be modelled as solid spheres, resulting in particles with a diameter of ca. 28 nm, suggesting $T = 3$ symmetry (Fig. S10b). For 24HB, a cylinder is chosen as its geometrical representative, resulting in a radius of 7.7 nm (Fig. S10c). The cylinder model additionally includes fitting to the Lorentzian peak (1), which has its center at 0.157 Å^{-1} corresponding to an inter-helical distance of 2.7 nm. Looking at the components' contributions, the Lorentzian peak (orange) accounts for the flattening of the cylinder model (green). As observed in TEM, two different populations, namely the complexed 24HB-2.5k structures and sphere-like assemblies, are assumed to be present in the complexed sample. Subsequently, the intensity distribution of the capsids is reduced to a form factor by subtraction from the 24HB-2.5k, thus features of the complexed 24HB become visible, and the complexed structures can be modeled as core-shell cylinders (main article Fig. 4d, Fig. S10d).

In order to avoid the subtraction factor, the system was furthermore modelled as a three-function model consisting of a core-shell cylinder for 24HB-2.5k, a sphere resembling icosahedral CP assemblies and the debye background (Fig. S10e). The model agrees well with the TEM measurements, suggesting diameters of 23.4 nm for 24HB-2.5k and 27.6 nm for spherical assemblies with $T = 3$ symmetry. The thickness of the CP shell on 24HB-2.5k is 5.1 nm. Furthermore, the impact of the single components on the final intensity distribution can be shown with this approach (Fig. S10f). The cylinder length, which is expected to be ca. 110 nm, has no impact on the model.

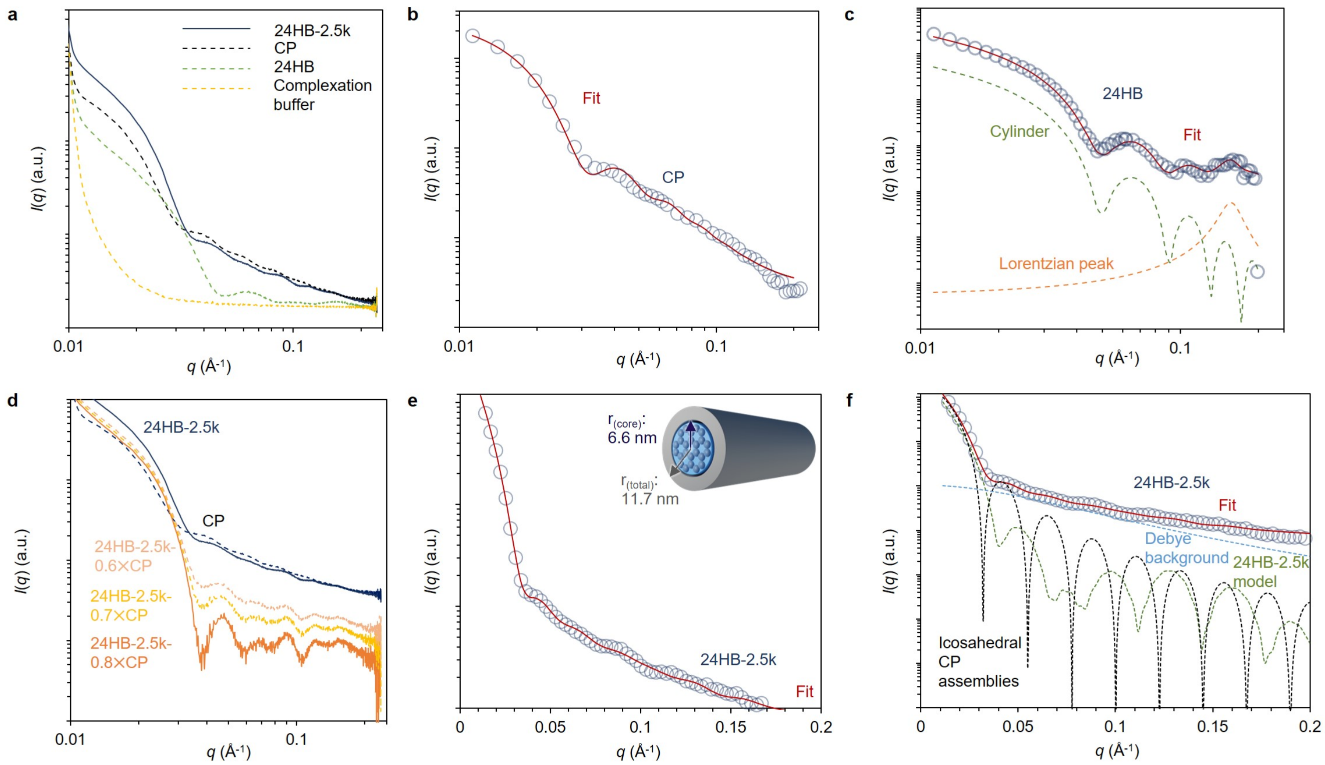

Fig. S10 a, Intensity distributions before buffer substraction for 24HB-2.5k (single protein layer, black), CPs only (dark blue dashed), 24HB (green dashed) and the complexation buffer (yellow dashed). The intensity distributions **b**, for sphere-like CP assemblies which are modelled as spheres and **c**, for 24HB a cylinder (green dashed) is used as geometric model. The fit is corrected by including the Lorentzian peak (orange dashed). **d**, Influence of the factor used for subtraction of the capsid scattering pattern from the pattern of the complexed structure (dark blue) through which features of 24HB become visible. **e**, 24HB-2.5k (blue circles, after background substraction) is modelled (red) using a three component system consisting of core-shell cylinder, spheres and a debye background. **f**, Breakdown of **e** showing the impact of the single components on the final model (red). Icosahedral CP assemblies (black) are represented by spheres and 24HB-2.5k by a core-shell cylinder (green).

Note S10: Negative-stain TEM images

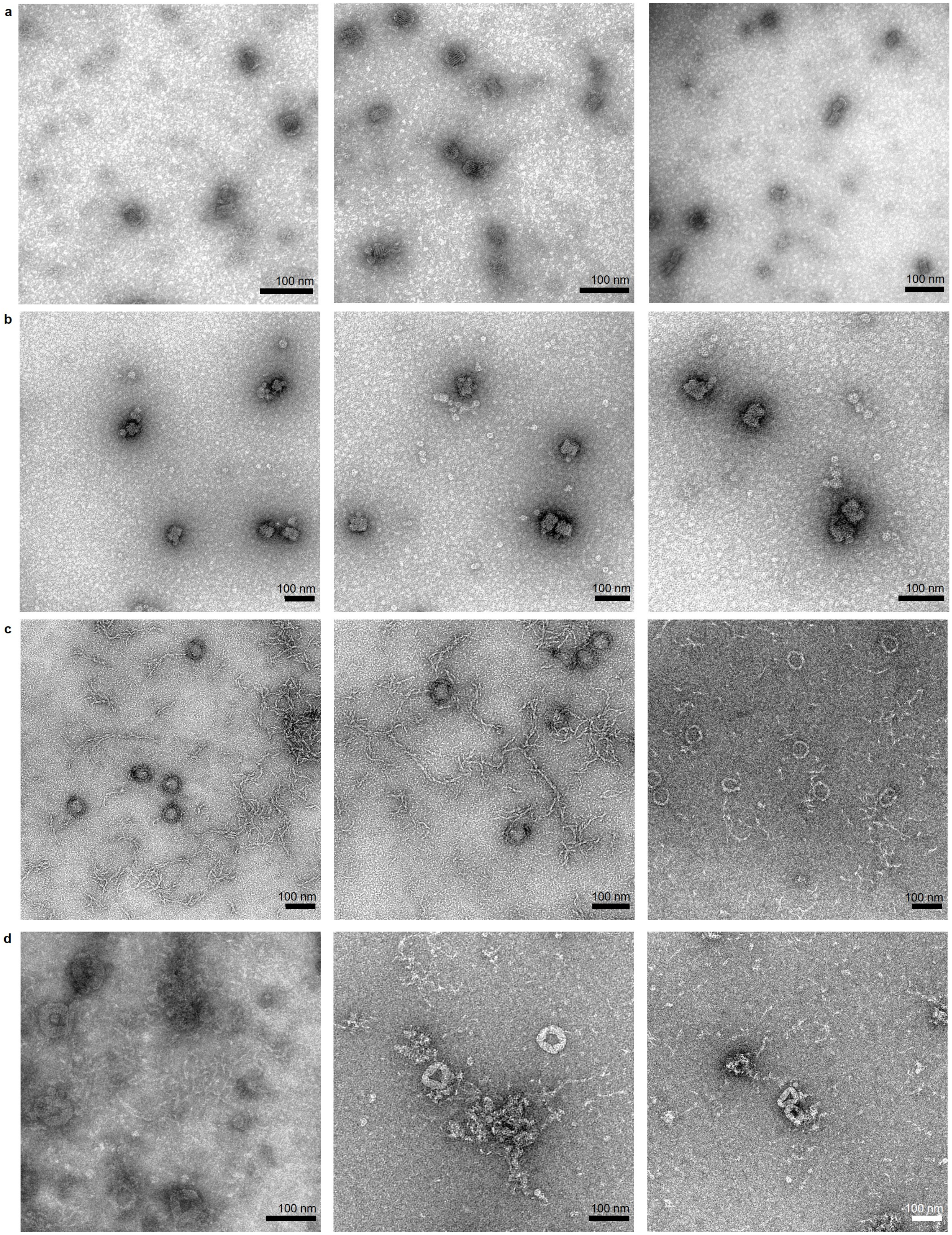

Fig. S11 Negative-stain TEM images showing origami structures complexed with CPs for full sample representation. 60HB with **a**, ε = 2k and **b**, ε = 10k. 13HR with **c**, ε = 2k and **d**, ε = 10k.

Note S11: Dimensions of coated structures

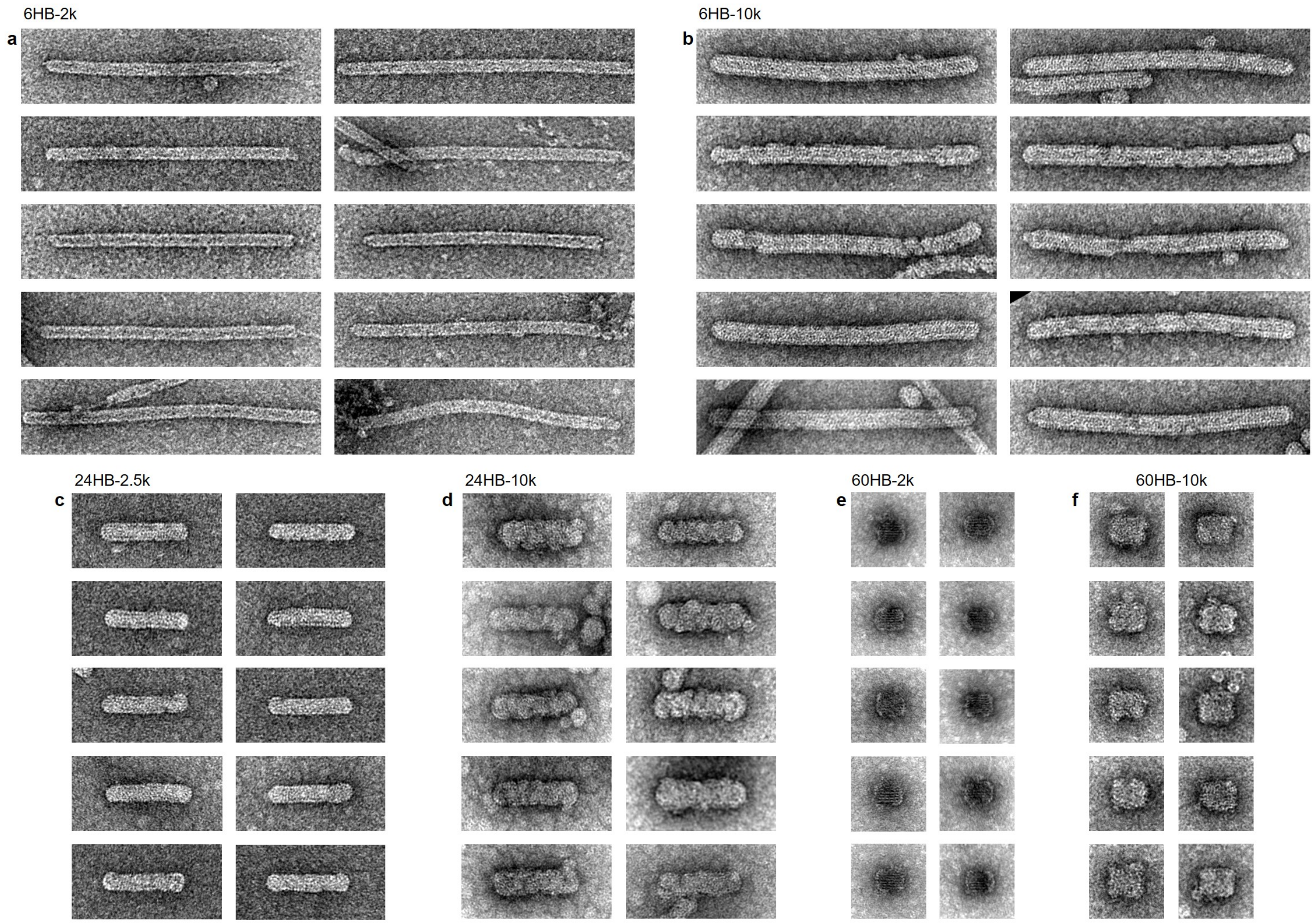

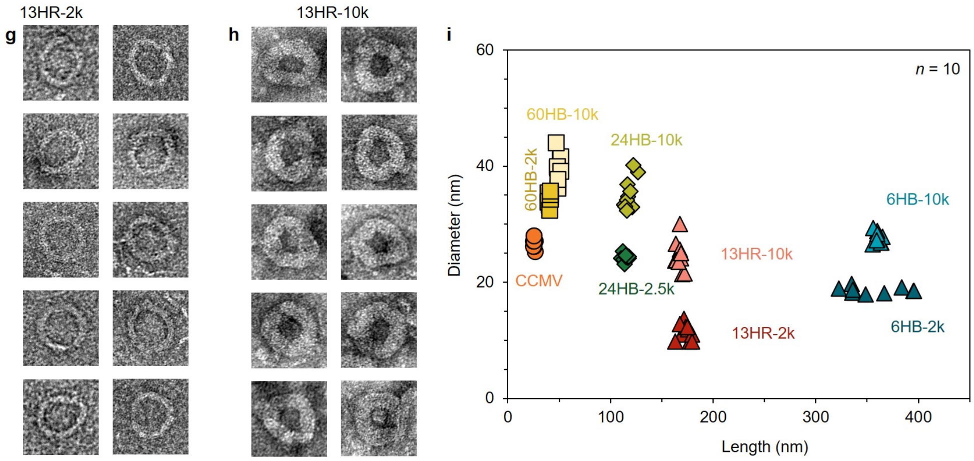

Sample	Length	Diameter
**CCMV**	26.1 ± 0.6 nm	26.7 ± 0.9 nm
**6HB-2k**	355.7 ± 27.3 nm	18.6 ± 0.5 nm
**6HB-10k**	360.4 ± 3.2 nm	27.7 ± 0.9 nm
**24HB-2.5k**	114.7 ± 2.4 nm	24.3 ± 0.6 nm
**24HB-10k**	118.3 ± 4.1 nm	35.2 ± 2.7 nm
**60HB-2k**	40.9 ± 1.1 nm	34.3 ± 1.0 nm
**60HB-10K**	49.8 ± 1.6 nm	39.5 ± 2.4 nm
**13HR-2k**	173.2 ± 5.3 nm	11.4 ± 1.4 nm
**13HR-10k**	167.2 ± 2.9 nm	24.5 ± 2.5 nm

Fig. S12 Negative-stain TEM images of **a**, 6HB-2k, **b**, 6HB-10k, **c**, 24HB-2.5k, **d**, 24HB-10k, **e**, 60HB-2k, **f**, 60HB-10k, **g**, 13HR-2k, and **h**, 13HR-10k. The image height corresponds in all images to 100 nm. **i**, Plot (left) and list (right) of the dimensions (length and diameter ± s.d.) of the coated structures shown in a-h.

Note S12: Coating of the nanocapsule structure

For initially testing the complexation between CPs and the nanocapsule, the permanently closed version of the structure was used. EMSA (Fig. S13a) suggests successful complexation, which was confirmed by negative-stain TEM (Fig. S13b, nanocapsule-2k). Similar to 6HB and 24HB, fully coated structures were observed at $\varepsilon \geq 2$k. However, when complexing the pH-responsive nanocapsule, pH changes should be avoided. The pH of the complexation buffer, pH 7.3, is exactly in the transition region between closed and open conformation of the nanocapsule (5). Hence, the complexation method was slightly modified, including the transfer of the purified origami structure into 6.5 mM HEPES buffer, pH 6.0. For the complexation, the CPs were titrated into the DNA origami solution in 1 μL steps, together with 0.4 μL of 25 mM acetic acid. Comparing the EMSA profile obtained from complexation at pH 6 (Fig. S13c), retention in the wells is observed already at $\varepsilon = 750$. Successful complexation was confirmed by negative-stain TEM (Fig. S13d).

For the release of the nanocapsule, heparin was used as a competitive agent to disassemble the CP coating. The amount of heparin used is expressed as the ratio between n_{sulfates} originating from heparin and $n_{\text{phosphates}}$ originating from the DNA backbone. A heparin molecule was estimated to contain on average 71 negatively charged sulfate groups, assuming an average molecular weight of 17–19 kDa and an average of 2.33 sulfate groups per repeating IdoA(2S)-GlcNS(6S) disaccharide unit (6). The total number of negative charge for the nanocapsule was estimated to be 17459. The release is monitored by EMSA (Fig. S13e), for the final experiments, a ratio of 1.9 was used, and the heparin was incubated with the structure for 10 min before pH adjustment.

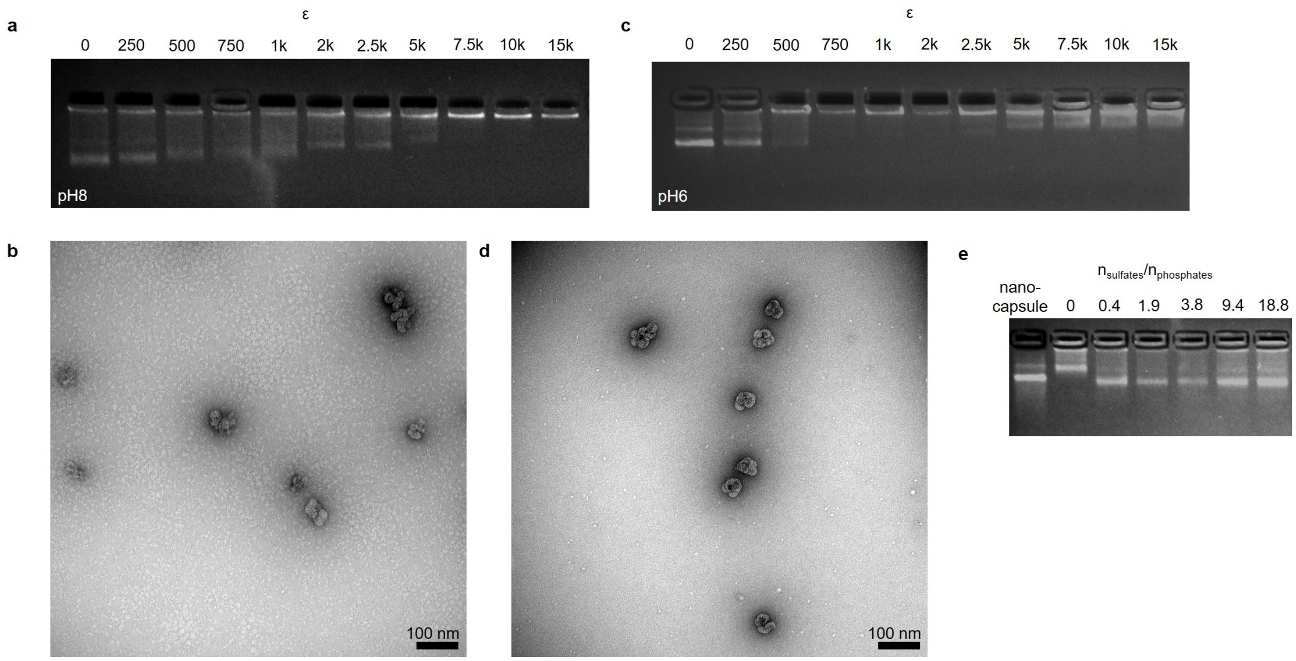

Fig. S13 a, EMSA of a permanently closed capsule complexed with CPs at physiological pH. **b**, Negative-stain TEM image showing the nanocapsule at ε = 2k. **c**, EMSA of the pH-responsive capsule complexed with CPs at pH 6. **d**, Negative-stain TEM image of the nanocapsule coated at pH 6, ε = 750. **e**, EMSA showing the release of the coated nanocapsule (ε = 750) at pH 6 using heparin as the competitive agent.

Note S13: Functionalization of 6HB

6HB was functionalized with AuNPs in a two-pot reaction, similar to previously described procedures (7, 8). First, the 6HB structure was prepared as described in the Method section, however, three staple strands were exchanged to contain an overhang which can later hybridize with oligonucleotide-functionalized AuNPs. After purification using poly(ethylene glycol) (PEG) precipitation (as described in Supplementary Note S20, similar to (9)), the folded structures were mixed with oligonuceotide-functionalized AuNPs which were added in 30× excess (10× excess per annealing site), heated to 40 °C and subsequently the temperature was decreased to 20 °C (–0.1 °C min^{-1} ramp).

Briefly, oligonucleotide-functionalized AuNPs were obtained by mixing 40 μL of AuNPs (5 nm diameter, citrate stabilized, 100 nM, Sigma Aldrich) with 0.8 μL of sodium dodecyl sulfate (SDS) for 20 min, before incubation with 4 μL thiol-modified oligonucleotides (for hybridizing with staple overhangs) for 30 min. The AuNPs were salt-aged using 2.5 M NaCl by 6× addition of 0.4 μL, 6× addition of 0.8 μL, 5× addition of 1.6 μL and a final addition of 2 μL. The interval between the additions is 5 min and all steps are performed at 40 °C and 600 rpm (Eppendorf ThermoMixer C). Subsequently, 60 μL of 1× folding buffer (FOB, 1× Tris-acetate-EDTA (TAE), 12.5 mM $MgCl_2$) supplemented with 0.02 % SDS are added and the incubation was continued for 1 h before the temperature was decrease to 20 °C for an overnight incubation.

Before usage, the oligonucleotide-functionalized AuNPs were purified from excess oligonucleotides using spin-filtration. After an initial washing step with 200 μL of the desired buffer (14,000 g, 5 min), 360 μL of AuNPs were added to the filter together with 120 μL 1× FOB with 0.02 % SDS and centrifuged for 10 min at 14,000 g, followed by a 3× addition of 200 μL of 1× FOB with 0.02 % SDS (10 min, 14,000 g). The purified DNA-functionalized AuNPs were recovered by inverting the filter and centrifugation for 2.5 min at 1,000 g.

The folded structures were purified from excess staple strands and AuNPs by PEG precipitation (10). Per 50 μL of folding reaction, 12.5 μL of PEG buffer containing 17.5 % (w/v) PEG8000, 1× TAE buffer, 500 mM NaCl and 10 mM $MgCl_2$ are used. Before centrifugation at 4 °C and 12,600 g for 30 min, the mixture is incubated at 4 °C for 10 min. The supernatant is discarded and the pellet resuspended in 1× FOB with 0.02 % SDS and incubated overnight at 30 °C, 600 rpm on an Eppendorf ThermoMixer C before the procedure is repeated to ensure full removal of excess AuNPs.

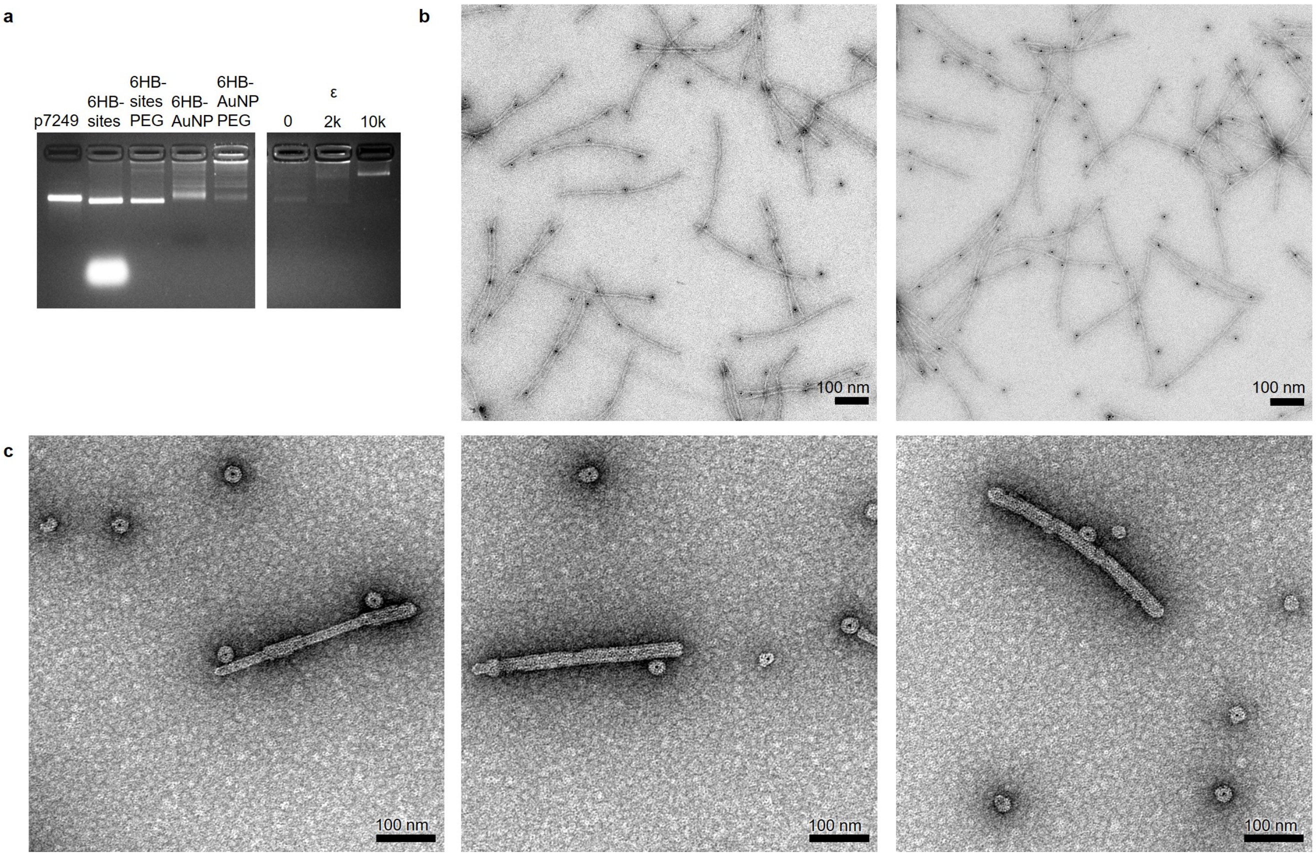

Fig. S14 a, Folding and purification of AuNP-functionalized 6HB (left). After purification (lane 3), the staples after the first folding step of 6HB containing AuNP annealing sites (lane 2) have been removed. Subsequently, AuNPs are annealed onto the structure (lane 4, excess gold is represented by the faster migrating, darker band) and in a two-step procedure purified (lane 5). As a reference, the scaffold is shown in lane 1. Complexation of functionalized 6HB with CPs (right). Negative-stain TEM images of **b**, the plain structures in 1× FOB which are buffer-exchanged and coated with CP at **c**, ε = 10k.

Note S14: DNase I digestion studies

Heparin was used as a competitive agent for the disintegration of CPs from the complexed structures resulting in plain origami structures to show the structural intactness after incubation with DNase I. As described in Note S12, the amount of heparin used is expressed as the ratio between n_{sulfates} and $n_{\mathrm{phosphates}}$. 6HB origami was estimated to have in total 14569 phosphate groups (7) while 24HB has 15504. For the final digestion experiments, 3.8$\times$ excess and 200$\times$ excess were used for structures coated with one or two CP layers, respectively.

The stability of the structures was furthermore tested in cell medium (Dulbecco's Modified Eagle Medium) supplemented with 5-10 % FBS, which was mixed with the complexed samples in a 1:1 ratio, resulting in an origami concentration of 3.2 nM (Fig. S15). After 24 h incubation at 37 °C, plain 6HB (left) was found to be partially digested in both FBS concentrations, while complexed structures (6HB-2k, right) were intact. The significant amount of 6HB remaining coated, and therefore remaining in the wells upon disassembly with heparin, could be explained by the large amount of protein present in FBS, which might unspecifically interact with heparin.

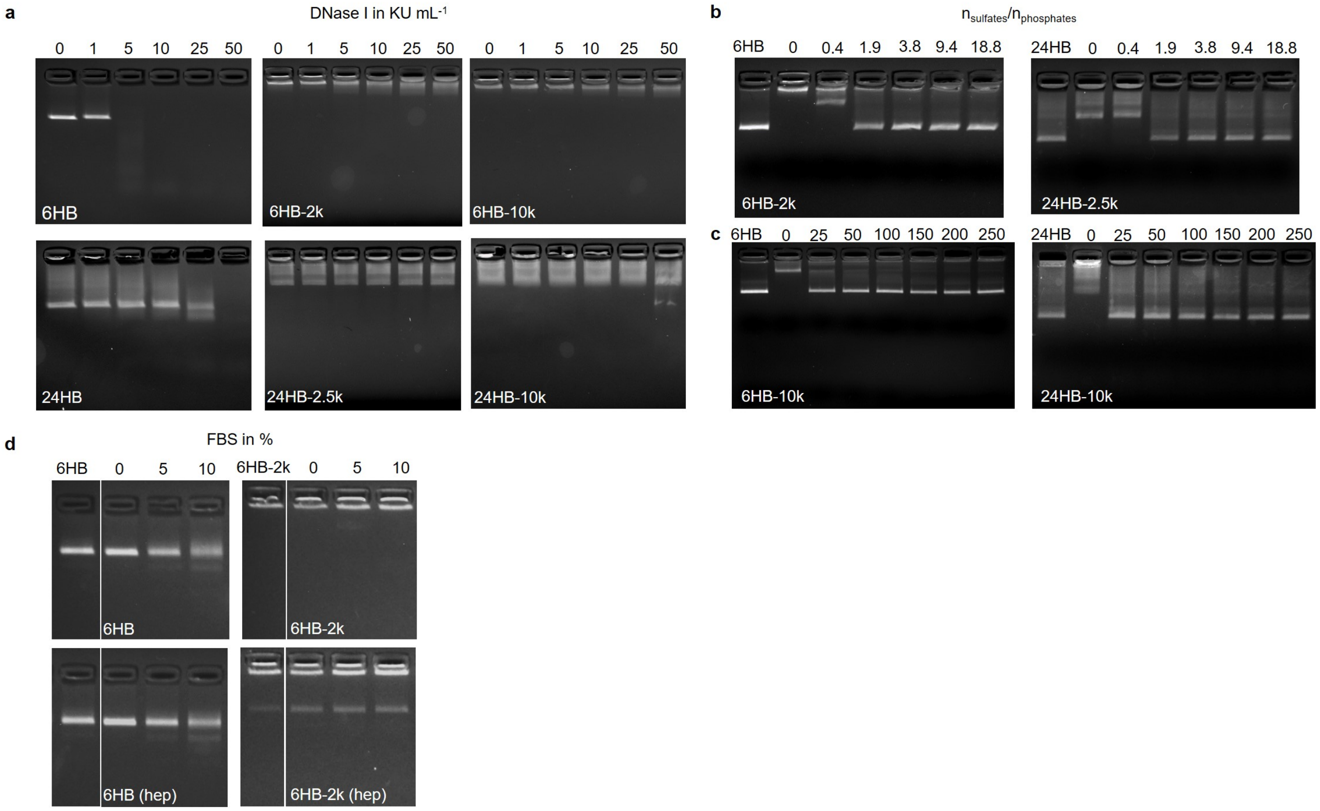

Fig. S15 a, Stability against DNase I of 6HB (top) and 24HB (bottom): Plain structures (left) are compared with structures coated with a single (middle) or two protein layers (right) before disassembly of the coating by the addition of heparin. **b**, Excess of heparin required for the release of the 6HB (left) and 24HB (right) when originally complexed with either ε = 2k or ε = 2.5k. **c**, Excess of heparin required for the release of the 6HB (left) and 24HB (right) when originally complexed with ε = 10k. **d**, Stability of 6HB (left) and 6HB-2k (right) upon 24 h-incubation in cell medium supplemented with different amounts of FBS. To decrease the retention in the wells, the coating has been removed with heparin (bottom).

Note S15: RNA-DNA hybrid origami

For the folding of the hybrid structure, the folding conditions have been optimized to ensure a high folding yield. Although a known thermal annealing program was used (11), different salt supplements in the folding buffer (1× TAE) were studied. EMSA of unpurified structures (Fig. S16a) shows the formation of double bands with increasing $MgCl_2$ concentrations. At 12.5 mM $MgCl_2$, the double band disappears, however the structures tend to aggregate in the wells. To prevent aggregation NaCl was added, leading to the final salt supplements of 5 mM $MgCl_2$, 1 mM NaCl. The double band is suggested to represent a dimer band, which is also reflected in the size distribution of both plain (blue) and complexed (RNA-6HB-500, grey) structures (Fig. S16c). Moreover, the poly(A) tail of the structure remains unfolded (i.e. no hybridization with staple strands), which additionally contributes to the heterogeneity of the complexed sample (Fig. S16b,c).

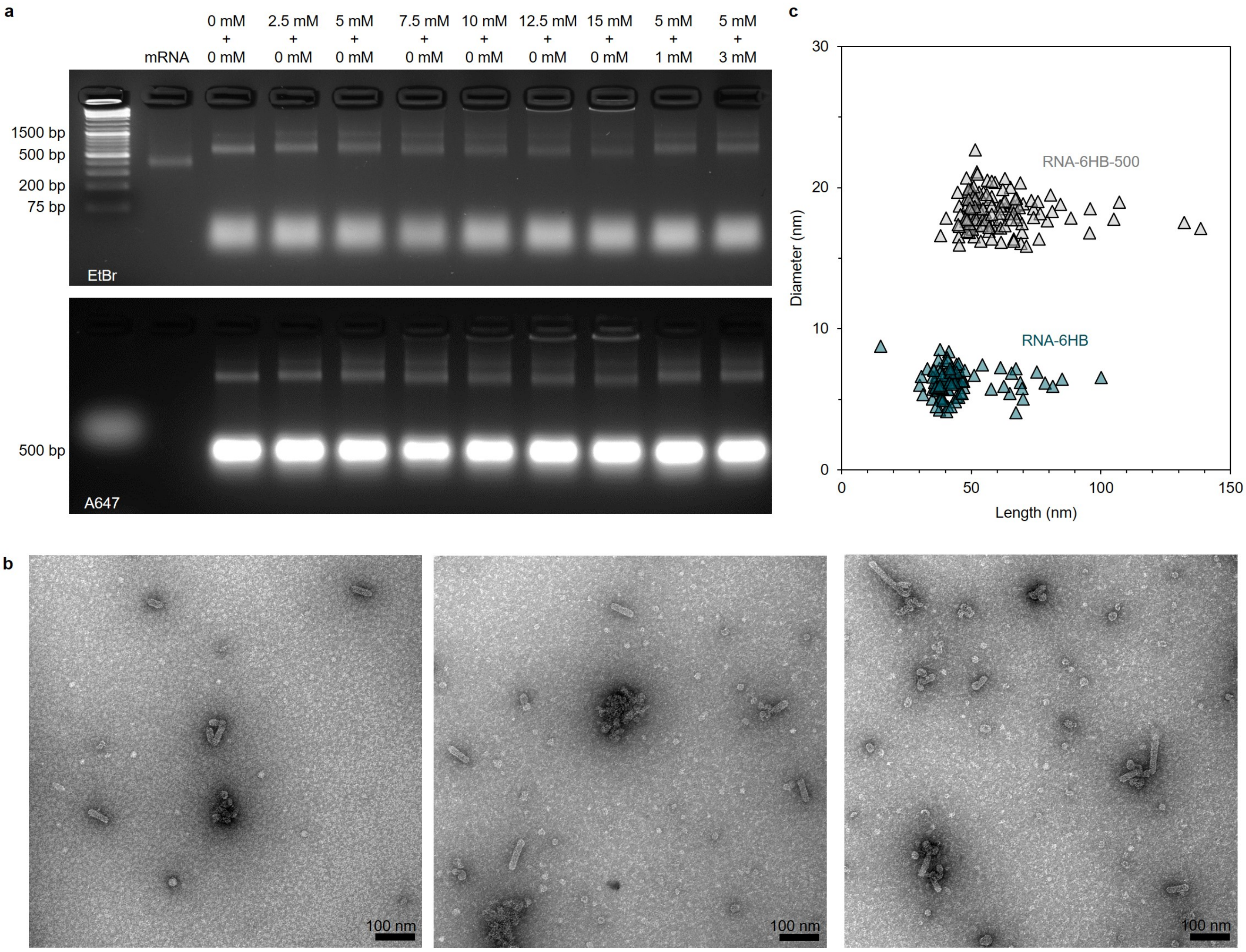

Fig. S16 a, Optimization of the folding conditions of RNA-6HB. Both the $MgCl_2$ and NaCl concentrations have been adjusted in the FOB (given as [$MgCl_2$] + [NaCl]). The folding was assessed from the EtBr (top) and A647 (bottom) channel. **b**, Negative-stain TEM images for RNA-6HB-500. **c**, Size distribution of plain RNA-6HB (blue) and RNA-6HB-500 (grey) showing an increase in both diameter and length upon complexation with CPs.

Note S16: Coating with norovirus (NoV)

The dissociation and reassembly of NoVLPs from different strains has been reported to be highly dependent on the ionic strength and pH of the buffer (12, 13). For instance, higher order oligomers, like 60- and 80mers have been found at high ionic strength and alkaline pH. Such oligomers were suggested to form due to a transition of the VP1, in which 180mers corresponding to the native virus particle disassemble into dimers followed by spontaneous reassembly. Furthermore, a dependency between oligomer formation and VP1 concentration was reported (13). We used 50 mM Tris-buffer, pH 8.9 for the disassembly of intact VLPs (Fig. S17a), which resulted predominantly in oligomers (Fig. S17b). To study reassembly on 6HB, the DNA origami was first mixed with VLPs at different concentrations ($\varepsilon = 500$ and 2k) and then dialyzed against the disassembly buffer. The same sample was then further dialyzed against reassembly buffer at low pH (sodium phosphate, pH 6), but no change in electrophoretic mobility is detected from EMSA (Fig. S17c). From negative-stain TEM a clear difference in the VP1 behaviour upon pH change can be observed. Compared to alkaline pH (Fig. S17d,e), VP1 is assembled to a larger extent into higher order oligomers at acidic conditions (Fig S17f).

NoV VP1 was described to lack the predominance of positively charged residues in the *N*-terminal region (14). Instead, the basic, minor structural protein VP2, most likely located in the capsid shell interior, is suggested to interact with nucleic acids (15). Since here, the VLPs are made entirely of VP1, we suggest ascribing the inability of ordered complex formation between VP1 and DNA origami to the lack of the RNA binding domain.

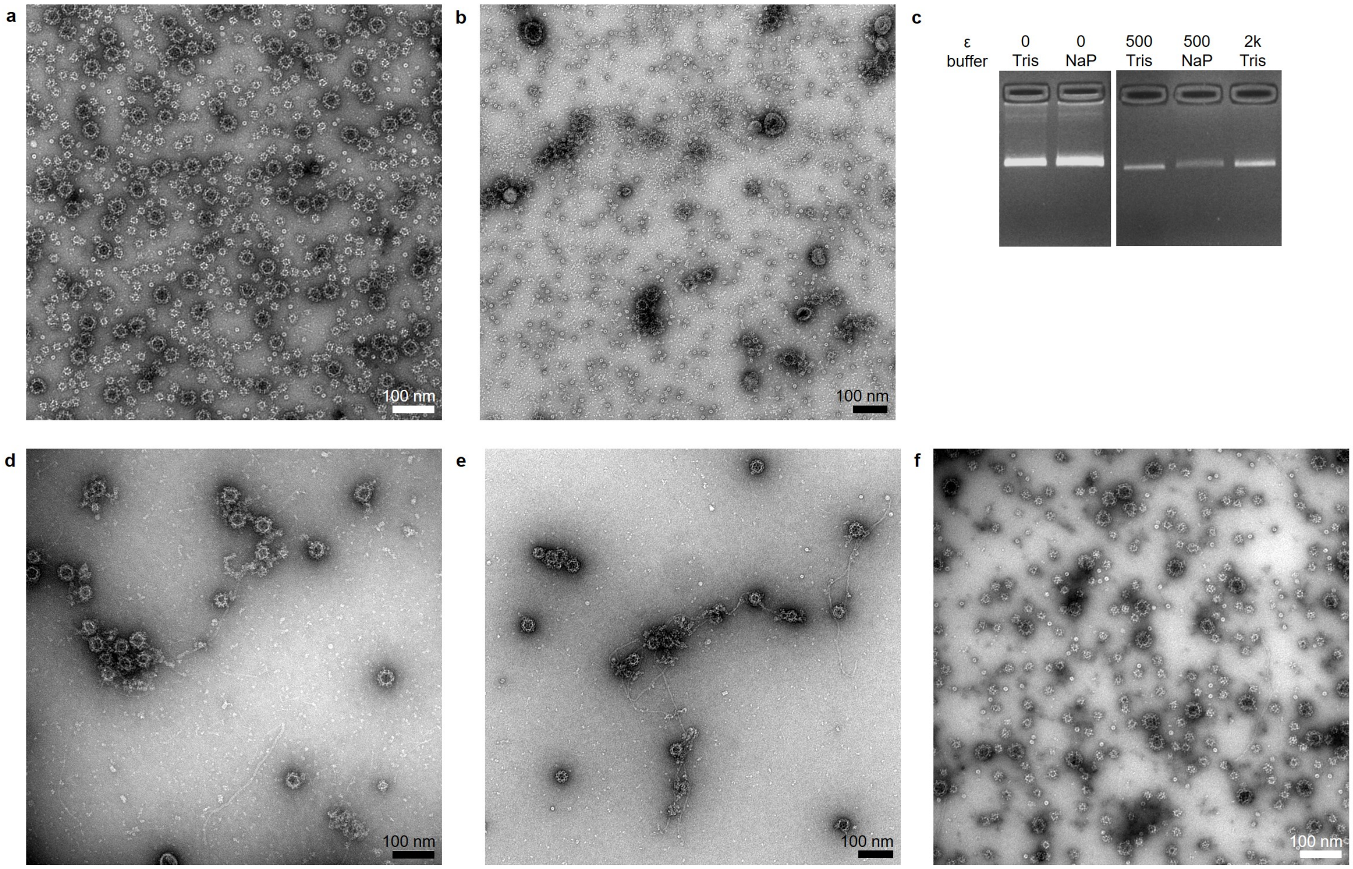

Fig. S17 Negative-stain TEM images of **a**, assembled and **b**, in 50 mM Tris-buffer, pH 8.9 disassembled NoVLPs. **c**, EMSA for 6HB at different ε after incubation with VP1. Negative-stain TEM images for **d**, 6HB-NoV-500, and **e**, 6HB-NoV-2k in 50 mM Tris-buffer, pH 8.9, and **f**, 6HB-NoV-500 in 100 mM sodium phosphate buffer, pH 6.

Note S17: Coating with simian virus 40 (SV40)

Disassembly of intact SV40 VLPs (Fig. S18a) results mainly in pentameric capsomers, as well as larger assemblies (Fig. S18b), which were not removed before complexation with 24HB. The EMSA (Fig. S18c) shows a small plateau in mobility decrease of the leading band around ε = 5k–7.5k, resulting in further analysis of 24HB-SV40-5k. In the absence of a template, the capsomers have been reported to assemble into tubular structures (16). A similar behaviour is also observed here (5k, Fig. S18d). However, in comparison to capsomers only, the diameter of the templated elongated structures (24HB-SV40-5k, Fig. S18e) is increased. The complexed structures can be classified into three groups, with assemblies around 100 nm being the majority. The shorter assemblies arise most likely from a heterogeneous template (truncated versions of ca. 30 nm and ca. 60 nm).

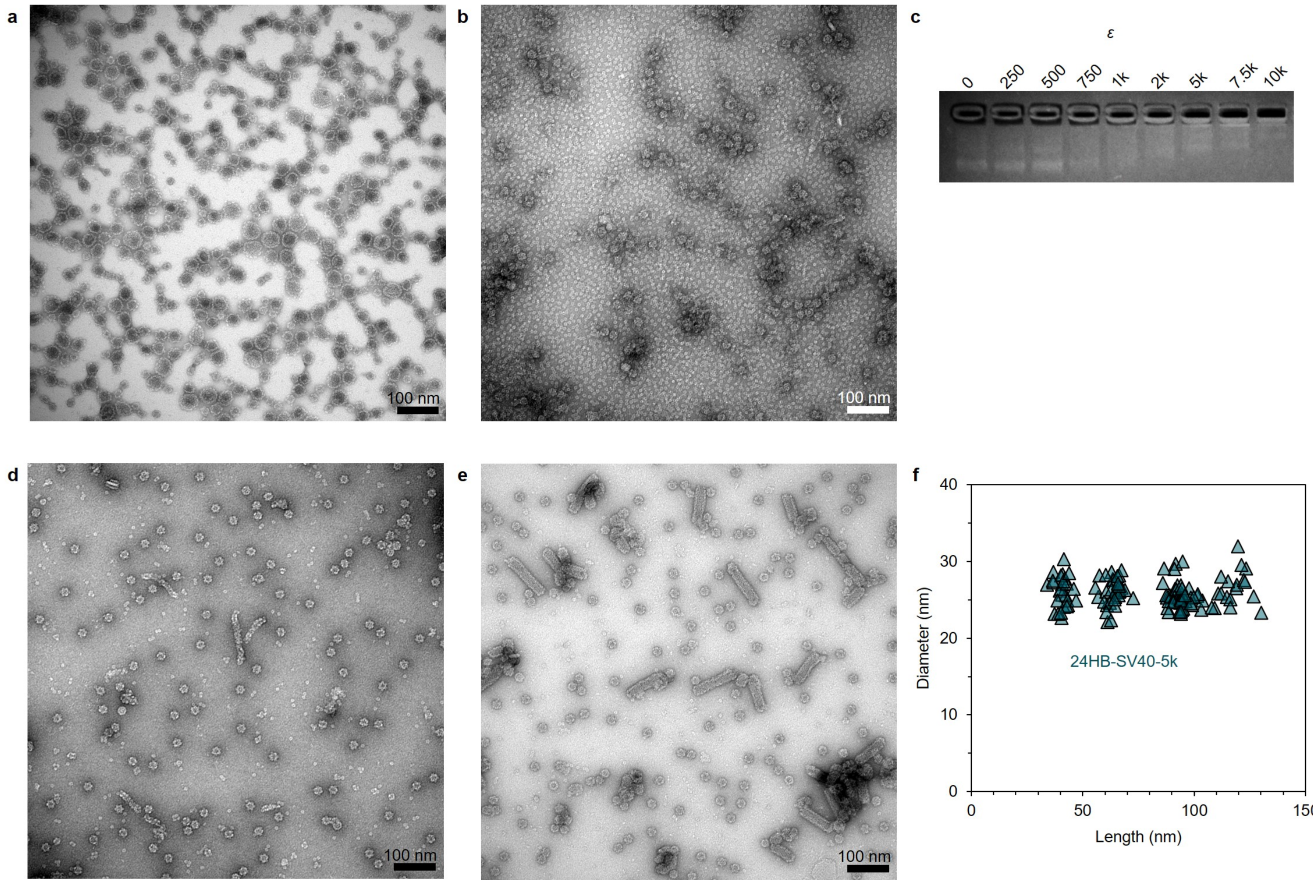

Fig. S18 Negative-stain TEM images of **a**, SV40 VLPs, and **b**, VLPs after disassembly, displaying VP1 being mainly present as pentamers. **c**, EMSA of 24HB shows a decrease in the mobility of the origami when incubated with increasing ε. Negative-stain TEM images of **d**, SV40 VP1 only, and **e**, 24HB-SV40-5k. **f**, Dimensions (length and diameter) of coated structures found in the 24HB-SV40-5k sample (n = 125).

Note S18: Coating with murine polyoma virus (MPyV)

The disassembled pentameric capsomers (main article Fig. 5j) were assembled either into VLPs (Fig. S19a) or complexed with both 6HB and 24HB. The EMSA shows a similar behaviour for 6HB (Fig. S19b, top) and 24HB (Fig. S19b, bottom). While partially coated structures are observed for 6HB-MPyV-750 (Fig. S19d), both structures are fully coated and display discrete sizes (Fig. S19c,e-f) at $\varepsilon > 1.25$k.

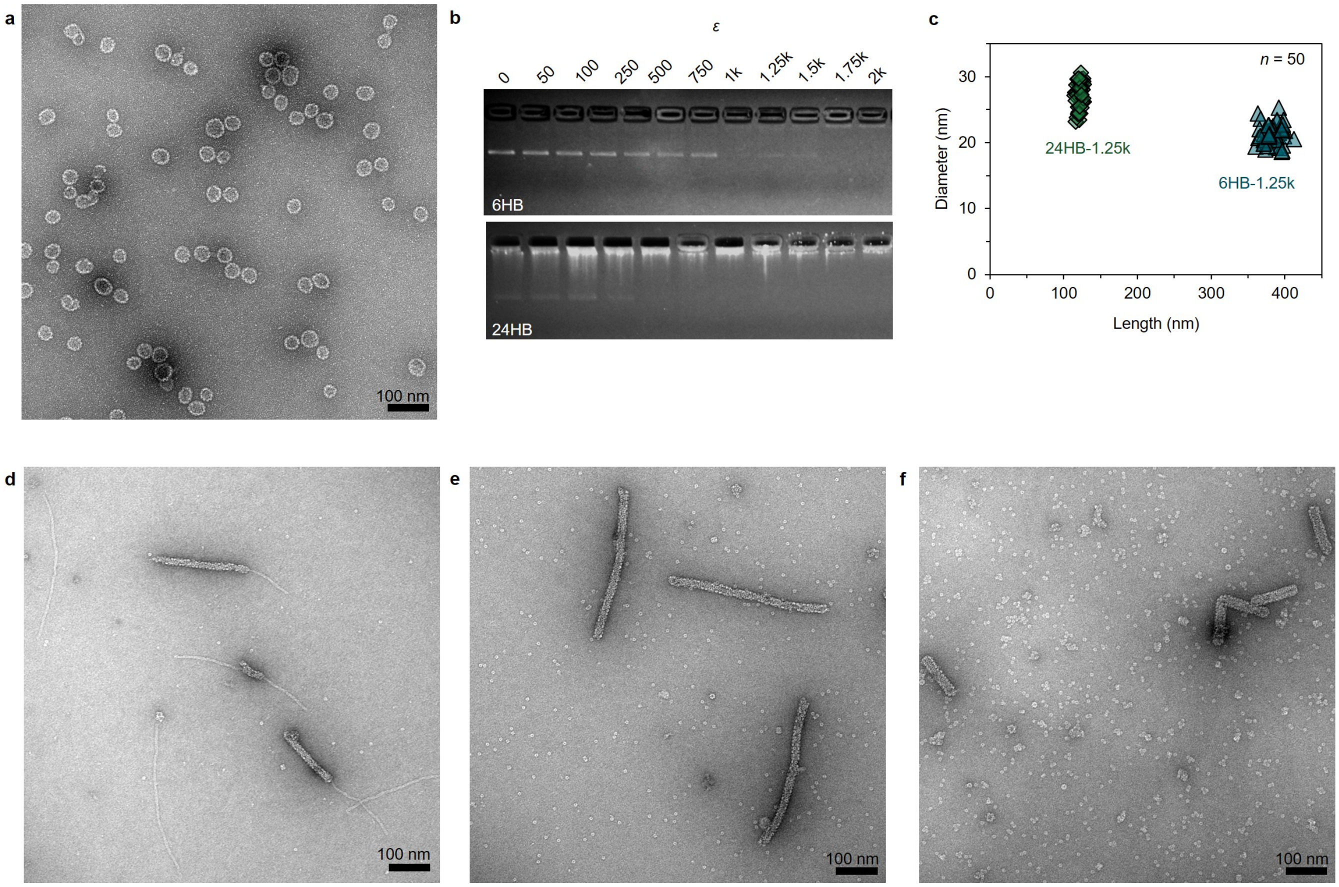

Fig. S19 a, Negative-stain TEM image of assembled VP1 VLPs. **b**, EMSA for 6HB (top) and 24HB (bottom) showing the decrease in mobility with increasing ε when incubated with VP1 pentamers. **c**, Evaluation of the dimensions (length and diameter) of coated 6HB (blue) and 24HB (green). Negative-stain TEM images for **d**, 6HB-MPyV-750, **e**, 6HB-MPyV-1.25k, and **f**, 24HB-MPyV-1.25k.

Note S19: Materials

All chemical reagents were obtained from commercial suppliers. 50× TAE buffer was purchased from Thermo Fisher Scientific, agarose from Biotop Oy, and ethidium bromide, heparin sodium salt from porcine intestinal mucosa, as well as DNase I from bovine pancreas from SigmaAldrich.

Note S20: Folding and Purification of DNA origami

For 6HB (17), 60HB (18), and 13HR (19) the p7249 scaffold was used. 24HB (20, 21), for one, was folded from p7560, and the nanocapsule (5) from p8064 (Supplementary Note S1).

Briefly, all structures with exception of 13HR and the nanocapsule were folded using final concentrations of 20 nM scaffold and 200 nM of each staple strand (20 nM scaffold and 150 nM staples for the nanocaspule) in a buffered environment. The FOB contains 1× Tris-acetate-ethylenediaminetetraacetic acid (EDTA) (1× TAE, 40 mM Tris, 20 mM acetic acid and 1 mM EDTA, pH 8.4) which was supplemented with varying salt concentrations depending on the DNA origami structure: 12.5 mM $MgCl_2$ for 6HB, 17.5 mM $MgCl_2$ for 24HB, 20 mM $MgCl_2$ with 5 mM NaCl for 60HB, and 15 mM $MgCl_2$ with 5 mM NaCl for the nanocapsule. The following thermal annealing ramp was used for 24HB, 60HB, and the nanocapsule folding: from 65 °C to 59 °C at a rate of –4 °C h^{-1}, and from 59 °C to 40 °C at –0.33 °C h^{-1}. For 6HB, a different annealing protocol was used: from 90 °C to 70 °C at a rate of −1.5 °C min^{-1}, from 70 °C to 60 °C at −0.75 °C min^{-1}, and from 60 °C to 27 °C at −3 °C h^{-1}.

Once the structures are folded, excess staple strands were removed using poly(ethylene glycol) (PEG) precipitation (9). After dilution in 1× FOB to a concentration of approx. 5 nM, the final volume was mixed in a 1:1 ratio with PEG precipitation buffer (1× TAE, 15 % (w/v) PEG8000, 505 mM NaCl) and centrifuged for 30 min at 14,000 *g*. The supernatant was removed, and the pelleted DNA origami resuspended in 1× FOB and incubated overnight at 30 °C, 600 rpm on an Eppendorf ThermoMixer C.

13HR was folded with final scaffold and staple concentrations of 10 nM and 50 nM, respectively, in 1× Tris-EDTA (1× TE pH 7.6, 10 mM Tris, 1 mM EDTA) buffer containing 10 mM $MgCl_2$. After 15 min incubation at 80 °C, the mixture was cooled from 79 °C to 71 °C at a rate of –1 °C min^{-1}, from 70 °C to 66 °C at –0.2 °C min^{-1}, from 65 °C to 60 °C at –2 °C h^{-1}, from 59 °C to 37 °C at –1 °C h^{-1}, from 36 °C to 30 °C at –4 °C h^{-1} and from 29 °C to 20 °C at –0.2 °C min^{-1}. Due to dimer formation and aggregation of 13HR, this structure was purified from an agarose gel. To this end, the samples were loaded on a 1 % (w/v) agarose gel (0.5 × Tris-borate-EDTA (TBE) buffer, pH 8.3, containing 44.5 mM Trizma base, 44.5 mM boric acid, 1 mM EDTA supplemented with 11 mM $MgCl_2$) and run for 2.25 h at 80 V. For visualization, EtBr (final concentration of 0.46 μg mL^{-1}) was used and the target band was cut out under ultraviolet (UV) light. The gel was cut in small pieces which were added into "freeze'n'squeeze" cups (Bio-Rad) and frozen for 5 min. Subsequently, the DNA origami was recovered after centrifugation at 16,000 *g* and 10 °C for 10 min.

The origami concentration was estimated according to Lambert-Beer's law from the absorbance measured at 260 nm (BioTek Eon Microplate Spectrophotometer, 2 μL sample volume, Take3 plate). The extinction coefficient of DNA origami structures is structure specific and it is estimated from the number of hybridized and non-hybridized nucleotides (22) (Table S1).

Table S1 Extinction coefficients at 260 nm for different DNA origami structures

**Name**	**Extinction coefficient (M^{-1} cm^{-1})**
6HB	0.98×10^8
24HB	1.076×10^8
60HB	0.91×10^8
13HR	1.3×10^8
Nanocapsule	1.047×10^8

Note S21: Isolation of native CCMV

Native CCMV particles were grown in and isolated from cowpea plants. Briefly, leaves of ten-day old plants were inoculated with CCMV, being either a suspension containing purified virus particles or infected cowpea leaves. After seven to ten days the plant material was harvested, homogenized in 0.2 M sodium acetate buffer, pH 4.8, supplemented with 0.01 M ascorbic acid and 0.01 M disodium EDTA, and pressed through a cheesecloth. After 1 h incubation at 4 °C, leaf tissue was pelleted by centrifugation at 10,000 rpm and 4 °C for 10 min. The CCMV containing supernatant was used to dissolve 10 % (w/v) solid PEG (MW = 6,000 g mol^{-1}) and CCMV was precipitated by centrifugation at 10,000 rpm and 4 °C for 15 min, after which it was resuspended in cold virus buffer (0.1 M sodium acetate, pH 5.0 supplemented with 1 mM EDTA and 1 mM sodium azide). Undissolvable material was removed by pelleting it at 10,000 rpm and 4 °C for 10 min. The virus particles were further purified by a density gradient centrifugation ($\leq$16 h, 40,000 rpm, 10 °C) using cesium chloride (37.5 % (w/w). The brownish, virus containing fraction was dialyzed against virus buffer (3 $\times$ 3 h, 4 °C) before isolating the CPs (23).

Note S22: Staple list for RNA-DNA hybrid origami

Table S2 Staple list for RNA-6HB. The strands marked with F have a sequence (underlined) complementary to the sequence of the ATTO590-strand and can be substitute with their corresponding strand to facilitate the integration of the fluorophore in the structure.

Number	Sequence (5'→ 3')
1	TGGTCGGACGCTGAGGTGGGCCAGGGCACCGCCCTCG
2	CTCGCCCTTGCTCACCATGG
3	GCAGCATATTTCTTCAAGAGGTACAGGTGCAAGGGAG
4	TTGCCGGTGGTGCAGATGAACT
5	TTGATGCCGTTCTTCTGCTTG
6	ACTTGAAGAAGTCGTGCTGCTTCAT
7	TTTGCTCAGGGCGGAGCAAGCCCCGCAGAAGGCAGCT
8	AGAAGAATGTAGTTGCCGTCGTGCACGC
9	GAAGATGGTGCGCTCCTGGACGCTTTATTC
10	CCGGCGGCGGTCACGAACTCCA
11	TAGGTGGCATCGCCCTCGCCCTCGCCGGACCGAGCTGC
12	AACTTCACCTCGGCGCGGGTCTGGGCATGG
13	GGATGTTGCCGTCCTCTCCAGCTTGTGCCCCA
14	GTGGTCGGGACCACCCCGGTGAACAGCTC
15	TCGGCCATGATATAGACGTTGTGGCTGTTCACC
16	TCAGGGTAGTTGTACCTTGAAGT
17	ACGCTGCCGTCCTCGATGTTGTGATCGCGCTTCTCGTTGGGGTC
18	GCCGTTTACGTCGCCGTCCAGCTCGACCAGTACGGGGC
19	CGTAGGTCAGGGTGGTCACGAGACTTGTG
20	CTTCCTACTCAGGTAGCCTTCGGGCATGGCGG
21	CTTGAAGTTGTCAGCTTGCCG
22	CCAGAAGCTGGGTGCTGGTAG
23	TGGCTCTGATGGGCGTAGCGGCTGAAGACTCCTTGAA
24	CGTCGCCGATGGGGGTGTTCTGCTCAGGTAGTGGTTGTCGGGCA
25	TTACTTGACCATGTGGCGGAT
26	GCAGGTACAGCTCGTCCATGCCGAGAGTGATC
27	CGATGCCCTTCAGCTCGATGCGGCGGCCGC
28	AAAGACCTTACTCTTCTTTTCTCTCTTATTTCCTC
29	TAATTAAGTTCACCAGGGTGTGGGCAGC
12F	AACTTCACCTCGGCGCGGGTCTGGGCATGGTTCGG TTT TGG GTT CCT G
19F	CGTAGGTCAGGGTGGTCACGAGACTTGTGTTCGG TTT TGG GTT CCT G
27F	CGATGCCCTTCAGCTCGATGCGGCGGCCGCTTCGG TTT TGG GTT CCT G
21F	CTTGAAGTTGTCAGCTTGCCGTTCGG TTT TGG GTT CCT G
ATTO590	/5ATTO590N/CAGGAACCCAAAACCG

Note S23: Recombinant expression and purification of MPyV capsomers

Wildtype VP1 proteins were recombinantly expressed in *E. coli* Rosetta (D3) pLysS cells (Novagen). To this end, a pGEX-4T-VP1 plasmid was constructed by inserting the VP1 gene into the pGEX-4T vector. The sequence encoding VP1 (M34958) (24) was amplified with 30 bp extensions homologous to the flanking vector to facilitate insertion into pGEX-4T (for sequences see Table S3) using an *in vivo* assembly method (25). For purification purposes, the vector contains a glutathione-S-transferase (GST) tag which is linked to the *N*-terminus of VP1 *via* a thrombin cleavage site. The plasmid was transformed into NEB 5-alpha High Efficiency Competent *E. coli* cells according to the manufacturer's instructions. Successful cloning was confirmed by colony polymerase chain reaction and Sanger sequencing after which the plasmids were transformed into Rosetta cells by heat shock, as per manufacturer's instructions.

The expression was performed similar as reported by Chuan *et al.* (26) Briefly, a single colony was used to inoculate the starting culture (5 mL terrific broth (TB) media containing 12 g L^{-1} tryptone, 24 g L^{-1} yeast extract, 0.4 % (v/v) glycerol, 2.31 g L^{-1} KH_2PO_4, and 12.24 g L^{-1} K_2HPO_4, supplemented with 34 mg L^{-1} chloramphenicol and 100 mg L^{-1} ampicillin) which was incubated overnight at 30 °C and 180 rpm. The starting culture was diluted 100× into the main culture (500 mL TB media supplemented with 34 mg L^{-1} chloramphenicol and 100 mg L^{-1} ampicillin) and grown at 37 °C and 180 rpm until the optical density at 600 nm (OD_{600}) reached 0.5-0.6. Subsequently, the cells were cooled down in an ice bath and induced with 0.3 mM isopropyl β-D-thiogalactopyranoside (IPTG, 26 °C, 16 h). The cells were harvested by centrifugation for 15 min at 4,000 *g*, 4 °C and stored at –20 °C.

The cells were lysed by resuspending the cell pellets in ca. 40 mL of "storage buffer" containing 40 mM Tris, 200 mM NaCl, 1 mM EDTA, 5 % (v/v) glycerol, and 5 mM DTT, pH 8.0, before sonicating for 3×20 s bursts at 20 % output with 1 min pause on ice between each pulse, followed by centrifugation for 25 min at 25,000 *g* at 4 °C. The supernatant was filtered through a 0.45 μm syringe filter (Merck Millipore) before further purification using affinity (GST Trap FF 5 mL column, GE Healthcare) and size exclusion (Superdex 200 10/300 GL column, GE Healthcare) chromatography (ÄKTA Pure, Cytiva/ NGC Discover, Bio-Rad). After initial equilibration of the GST Trap FF column, the proteins were injected (0.5 mL min^{-1} flow rate) and the column was washed with "storage buffer". Subsequently, 5 mL of "storage buffer" containing 50-100 units of thrombin were manually loaded onto the column, and the column was sealed and incubated for 16 h at 4 °C to facilitate the cleavage of the VP1 from the column. VP1 was eluted with "storage buffer" and subsequently aggregates were removed by passing the sample through the size exclusion column (equilibrated with "storage buffer"). The purity of the sample was evaluated from SDS-PAGE (Mini-PROTEAN TGX Precast Protein Gels, Bio-Rad, 30 min, 200 V in 25 mM Tris, 190 mM glycine, 0.1 % SDS, Fig. S3). The concentration of the capsomers was determined based on their absorbance at 280 nm (extinction coefficient of the monomer of 57,870 M^{-1} cm^{-1}) using a BioTek Eon Microplate Spectrophotometer (2 μL sample, Take3 plate).

For *in vitro* reassembly of the purified capsomers into VLPs, the capsomers were dialysed overnight at room temperature against assembly buffer containing 0.5 M $(NH_4)_2SO_4$, 20 mM Tris, 5 % (v/v) glycerol, and 1 mM $CaCl_2$, pH7.4 using Slize-A-Lyzer Mini Dialysis cups (3.5 kDa MWCO, Thermo Scientific).

Table S3 Oligonucleotides used for cloning and PCR amplification and amino acid sequence of VP1. Underlining represents extensions with homology to flanking sequences for *in vivo* cloning.

Name	Sequence (5'→ 3')/(N-→C-terminus)
VP1-F	<u>ccaaaatcggatctggttccgcgtggatcc</u>gcccccaaaagaaaaagcgg
VP1-R	<u>atcaccgaaacgcgcgaggcagatcgtcag</u>ttaatttccaggaaatacagtctttgt
pGEX-F	ctgacgatctgcctcgcg
pGEX-R	ggatccacgcggaaccagatc
Wildtype VP1 (42.4 kDa)	APKRKSGVSKCETKCTKACPRPAPVPKLLIKGGMEVLDLVTGPDSVTEIEAFLNPR<br>MGQPPTPESLTEGGQYYGWSRGINLATSDTEDSPGNNTLPTWSMAKLQLPMLNED<br>LTCDTLQMWEAVSVKTEVVGSGSLLDVHGFNKPTDTVNTKGISTPVEGSQYHVFA<br>VGGEPLDLQGLVTDARTKYKEEGVVTIKTITKKDMVNKDQVLNPISKAKLDKDGM<br>YPVEIWHPDPAKNENTRYFGNYTGGTTTPPVLQFTNTLTTVLLDENGVGPLCKGEG<br>LYLSCVDIMGWRVTRNYDVHHWRGLPRYFKITLRKRWVKNPYPMASLISSLFNNM<br>LPQVQGQPMEGENTQVEEVRVYDGTEPVPGDPDMTRYVDRFGKTKTVFPGN

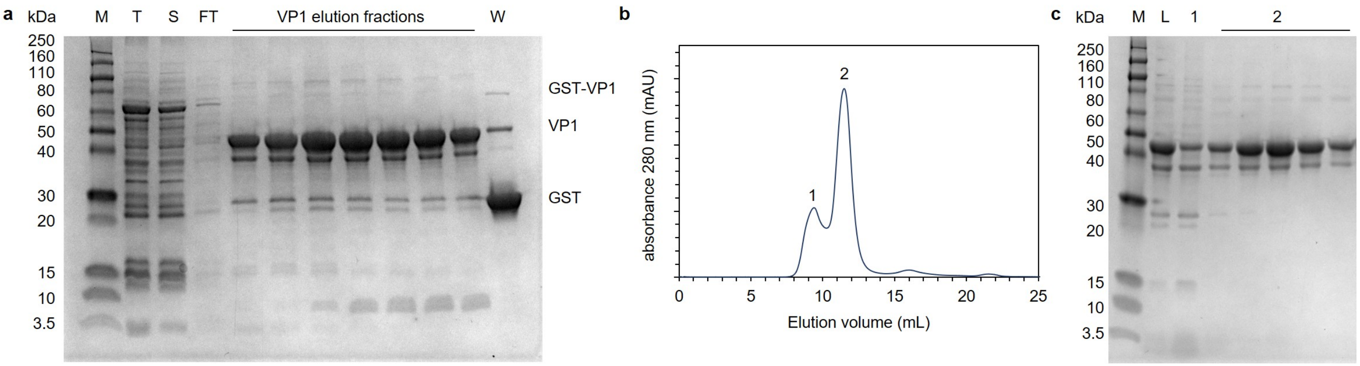

Fig. S20 a, SDS-PAGE comparing the proteins present in VP1 elution fractions after GST affinity chromatography with the total cell lysate (T), the soluble protein (S), the flow-through during sample loading (FT) and the wash fraction (W) eluting the cleaved GST. **b**, Size exclusion chromatogram of VP1 elution fractions, separating aggregates (peak 1) from the capsomers used for the experiments (peak 2). **c**, SDS-PAGE comparing the eluted fractions from size exclusion chromatography, 1 and 2, with the VP1 fraction from the affinity chromatography (L). The size marker is abbreviated with (M).

Note S24: Collection of parameters used in cryo-EM and single-particle reconstruction

Table S4 Cryo-EM structure determination parameters

	**6HB-2k** (EMD-16076)	**6HB-10k inner** (EMD-16077)	**6HB-10k outer** (EMD-16078)	**6HB-2k cap** (EMD-16079)	**24HB-2.5k** (EMD-16080)
**Data collection and processing**					
Magnification	150,000×	150,000×	150,000×	150,000×	150,000×
Voltage (kV)	200	200	200	200	200
Electron exposure (e^{-}/Å^2)	40	40	40	40	40
Defocus range (μm)	0.7–2.1	0.7–2.1	0.7–2.1	0.7–2.1	0.8–2.3
Pixel size (Å)	0.96	0.96	0.96	0.96	0.96
Symmetry imposed					
Point group	C1	C1	C1	C1	C2
Helical turn (°); rise (Å)	63.8; 15.4	63.8; 15.4	-107.1; 9.9	N/A; N/A	48.2; 23.4
Initial helical segments (no.)	837,120	384,254	32,473	3904	37,075
Final helical segments (no.)	695,465	32,473	6141	3740	4197
Map resolution (Å)	4.3	7.3	7.0	8.8	10.2
FSC threshold	0.143	0.143	0.143	0.143	0.143
Map sharpening B factor (Å^2)	-300	-300	-300	-300	-300

Table S5 Model refinement and validation

	**6HB-2k** (PDB:8BI4)
**Refinement & validation**	
Model-to-map resolution (Å)	4.4
FSC threshold	0.5
Model-to-map CC	
Main chain	0.79
Side chain	0.78
Model composition	
Chains	6
Non-hydrogen atoms	6600
Model resolution range (Å)	
B factors (Å^2)	
Protein	34.6
R.m.s deviations	
Bond lengths (Å)	0.004
Bond angles (°)	1.003
Validation	
MolProbity score	1.29
Clash score	2.11
Rotamer outliers (%)	0.14
Ramachandran plot	
Favored (%)	95.63
Allowed (%)	4.02
Outliers (%)	0.34

Supplementary Information references

1. Fischer, S.; Hartl, C.; Frank, K.; Rädler, J. O.; Liedl, T.; Nickel, B. Shape and Interhelical Spacing of DNA Origami Nanostructures Studied by Small-Angle X-ray Scattering. *Nano Lett.* **2016**, *16*, 4282–4287, https://doi.org/10.1021/acs.nanolett.6b01335.
2. Lázaro, G. R.; Dragnea, B.; Hagan, M. F. Self-assembly of convex particles on spherocylindrical surfaces. *Soft Matter* **2018**, *14*, 5728–5740, https://doi.org/10.1039/C8SM00129D.
3. Speir, J. A.; Munshi, S.; Wang, G.; Baker, T. S.; Johnson, J. E. Structures of the native and swollen forms of cowpea chlorotic mottle virus determined by X-ray crystallography and cryo-electron microscopy. *Structure* **1995**, *3*, 63–78, https://doi.org/10.1016/S0969-2126(01)00135-6.
4. Bancroft, J. B.; Hills, G. J.; Markham, R. A Study of the Self-Assembly Process in a Small Spherical Virus. Formation of Organized Structures from Protein Subunits *in Vitro*. *Virology* **1967**, *31*, 354–379, https://doi.org/10.1016/0042-6822(67)90180-8.
5. Ijäs, H.; Hakaste, I.; Shen, B.; Kostiainen, M. A.; Linko, V. Reconfigurable DNA Origami Nanocapsule for pH-Controlled Encapsulation and Display of Cargo. *ACS Nano* **2019**, *13*, 5959–5967, https://doi.org/10.1021/acsnano.9b01857.
6. Välimäki, S.; Khakalo, A.; Ora, A.; Johansson, L.-S.; Rojas, O. J.; Kostiainen, M. A. Effect of PEG-PDMAEMA Block Copolymer Architecture on Polyelectrolyte Complex Formation with Heparin. *Biomacromolecules* **2016**, *17*, 2891–2900, https://doi.org/10.1021/acs.biomac.6b00699.
7. Julin, S.; Nonappa,; Shen, B.; Linko, V.; Kostiainen, M. A. DNA-Origami-Templated Growth of Multilamellar Lipid Assemblies. *Angew. Chem. Int. Ed.* **2021**, *60*, 827–833, https://doi.org/10.1002/anie.202006044.
8. Ijäs, H.; Hakaste, I.; Shen, B.; Kostiainen, M. A.; Linko, V. Reconfigurable DNA Origami Nanocapsule for pH-Controlled Encapsulation and Display of Cargo. *ACS Nano* **2019**, *5*, 5959–5967, https://doi.org/10.1021/acsnano.9b01857.
9. Stahl, E.; Martin, T. G.; Praetorius, F.; Dietz, H. Facile and scalable preparation of pure and dense DNA origami solutions. *Angew. Chem. Int. Ed.* **2014**, *53*, 12735–12740, https://doi.org/10.1002/anie.201405991.
10. Shaw, A.; Benson, E.; Högberg, B. Purification of Functionalized DNA Origami Nanostructures. *ACS Nano* **2015**, *9*, 4968–4975, https://doi.org/10.1021/nn507035g.
11. Torelli, E.; Shirt-Ediss, B.; Navarro, S. A.; Manzano, M.; Vizzini, P.; Krasnogor, N. Light-up split Broccoli aptamer as a versatile tool for RNA assembly monitoring in cell-free TX-TL system, hybrid RNA/DNA origami tagging and DNA biosensing. *bioRxiv* **2022**, https://doi.org/10.1101/2022.07.20.500791.
12. White, L. J.; Hardy, M. E.; Estes, M. Biochemical Characterization of a Smaller Form of Recombinant Norwalk Virus Capsids Assembled in Insect Cells. *J Virol.* **1997**, *71*, 8066–8072, https://doi.org/10.1128/jvi.71.10.8066-8072.1997.
13. Shoemaker, G. K.; van Duijn, E.; Crawford, S. E.; Uetrecht, C.; Baclayon, M.; Roos, W. H.; Wuite, G. J. L.; Estes, M. K.; Prasat, B. B. B.; Heck, A. J. R. Norwalk Virus Assembly and Stability Monitored by Mass Spectrometry. *Mol. Cell Proteomics* **2010**, *9*, 1742–1751, https://doi.org/10.1074/mcp.M900620-MCP200.
14. Prasad, B. V. V.; Hardy, M. E.; Dokland, T.; Bella, J.; Rossmann, M. G.; Estes, M. X-ray Crystallographic Structure of the Norwalk Virus Capsid. *Science* **1999**, *268*, 287–290, https://doi.org/10.1126/science.286.5438.287.
15. Vongpunsawad, S.; Prasad, B. V. V.; Estes, M. Norwalk Virus Minor Capsid Protein VP2 Associates within the VP1 Shell Domain. *J Virol.* **2013**, *87*, 4818–4825, https://doi.org/10.1128/JVI.03508-12.
16. Asor, R.; Singaram, S. W.; Levi-Kalisman, Y.; Hagan, M. F.; Raviv, U. Effect of Ionic Strength on the Assembly of Simian Vacuolating Virus Capsid Protein Around Poly(Styrene Sulfonate). *bioRxiv* **2022**, https://doi.org/10.1101/2022.02.25.481942.
17. Bui, H.; Onodera, C.; Kidwell, C.; Tan, Y.; Graugnard, E.; Kuang, W.; Lee, J.; Knowlton, W. B.; Yurke, B.; Hughes, W. L. Programmable periodicity of quantum dot arrays with DNA origami nanotubes. *Nano Lett.* **2010**, *10*, 3367–3372.
18. Linko, V.; Shen, B.; Tapio, K.; Toppari, J. J.; Kostiainen, M. A.; Tuukkanen, S. One-step large-scale deposition of salt-free DNA origami nanostructures. *Sci. Rep.* **2015**, *5*, 15634.
19. Nguyen, M.-K.; Nguyen, V. H.; Natarajan, A. K.; Huang, Y.; Ryssy, J.; Shen, B.; Kuzyk, A. Ultrathin silica coating of DNA origami nanostructures. *Chem. Mater.* **2020**, *32*, 6657–6665.
20. Ijäs, H.; Shen, B.; Heuer-Jungemann, A.; Keller, A.; Kostiainen, M. A.; Liedl, T.; Ihalainen, J. A.; Linko, V. Unraveling the interaction between doxorubicin and DNA origami nanostructures for customizable chemotherapeutic drug release. *Nucleic Acids Res.* **2021**, *49*, 3048–3062, https://doi.org/10.1093/nar/gkab097.
21. Seitz, I.; Ijäs, H.; Linko, V.; Kostiainen, M. A. Optically responsive protein coating of DNA origami for triggered antigen targeting. *ACS Appl. Mater. Interfaces* **2022**, *14*, 38515–38524.
22. Hung, A. M.; Micheel, C. M.; Bozano, L. D.; Osterbur, L. W.; Wallraff, G. M.; Cha, J. N. Large-area spatially ordered arrays of gold nanoparticles directed by lithographically confined DNA origami. *Nat. Nanotechnol.* **2010**, *5*, 121–126.
23. Comellas Aragones, M. *The cowpea chlorotic mottle virus as a building block in nanotechnology*; ISBN: 9789090249339: Dissertation, RU Radboud Universiteit Nijmegen, 2010; https://hdl.handle.net/2066/74710.

24. Catrice, E. V. B.; Sainsbury, F. Assembly and Purification of Polyomavirus-Like Particles from Plants. *Mol. Biotechnol.* **2015**, *57*, 904–913, https://doi.org/10.1007/s12033-015-9879-9.
25. García-Nafría, J.; Watson, J. F.; Greger, I. H. IVA cloning: A single-tube universal cloning system exploiting bacterial *In Vivo* Assembly. *Sci Rep* **2016**, *6*, 27459, https://doi.org/10.1038/srep27459.
26. Chuan, Y. P.; Lua, L. H. L.; Middelberg, A. P. J. High-level expression of soluble viral structural protein in *Escherichia coli*. *J. Biotechnol.* **2008**, *134*, 64–71, https://doi.org/10.1016/j.jbiotec.2007.12.004.
